# Porous Electropolymerized Films of Ruthenium Complex: Photoelectrochemical Properties and Photoelectrocatalytic Synthesis of Hydrogen Peroxide

**DOI:** 10.3390/molecules29030734

**Published:** 2024-02-05

**Authors:** Hong-Ju Yin, Ke-Zhi Wang

**Affiliations:** 1 Beijing Key Laboratory of Energy Conversion and Storage Materials, College of Chemistry, Beijing Normal University, Beijing 100875, China; yinhongju411@163.com; 2 College of Chemistry and Environmental Science, Qujing Normal University, Qujing 655011, China

**Keywords:** ruthenium complex, photoelectrochemical property, hydrogen peroxide, modified electrode, photoelectrochemical oxygen reduction, electropolymerization

## Abstract

The photoelectrochemical cells (PECs) performing high-efficiency conversions of solar energy into both electricity and high value-added chemicals are highly desirable but rather challenging. Herein, we demonstrate that a PEC using the oxidatively electropolymerized film of a heteroleptic Ru(II) complex of [Ru(bpy)(**L**)_2_](PF_6_)_2_ **Ru1** {bpy and **L** stand for 2,2′-bipyridine and 1-phenyl-2-(4-vinylphenyl)-1*H*-imidazo[4,5-*f*][1,10]phenanthroline respectively}, poly**Ru1**, as a working electrode performed both efficient in situ synthesis of hydrogen peroxide and photocurrent generation/switching. Specifically, when biased at −0.4 V vs. saturated calomel electrode and illuminated with 100 mW·cm^−2^ white light, the PEC showed a significant cathodic photocurrent density of 9.64 μA·cm^−2^. Furthermore, an increase in the concentrations of quinhydrone in the electrolyte solution enabled the photocurrent polarity to switch from cathodic to anodic, and the anodic photocurrent density reached as high as 11.4 μA·cm^−2^. Interestingly, in this single-compartment PEC, the hydrogen peroxide yield reached 2.63 μmol·cm^−2^ in the neutral electrolyte solution. This study will serve as a guide for the design of high-efficiency metal-complex-based molecular systems performing photoelectric conversion/switching and photoelectrochemical oxygen reduction to hydrogen peroxide.

## 1. Introduction

The conversion of solar energy into both electricity and high-value-added chemicals presents a promising sustainable energy technique [1]. A photoelectrochemical cell (PEC), would provide a simple and efficient means to evaluate materials and establish a structure–performance relationship for the above-mentioned purposes [2]. To do so, the stable immobilization of functional molecules on electrodes is particularly important. Among the main electrode modification techniques reported, including electrospray [3], covalent self-assembly [4], electropolymerization [2], layer-by-layer assembly [5], spin coating and drop casting [6], electropolymerization could quickly separate polymers electrodeposited on electrodes from their precursor monomers in the electrolyte solution, and the thickness of the deposited films can be controlled by adjusting electropolymerization parameters or monomer concentrations [7].

On the other hand, substantial efforts have focused on the solar-energy splitting of water into hydrogen and oxygen, and carbon dioxide reduction into high-value chemicals [8]. In contrast, the photoelectrochemical conversion and storage of solar energy in the form of hydrogen peroxide (H_2_O_2_) have received less attention [9,10,11]. H_2_O_2_, a green chemical oxidant and an ideal energy storage medium, which is growing in demand year by year, is widely utilized in various fields, including fuel cells, industrial manufacturing, organic synthesis, and medical treatment [11]. H_2_O_2_ is mainly synthesized industrially by the anthraquinone method [12,13]. This method requires precious palladium catalysts, complex steps to extract H_2_O_2_ from organic solvents, a large amount of energy input, and the production of a substantial amount of organic byproduct waste. Comparatively, the photoelectrosynthesis of H_2_O_2_ via the selective two-electron oxygen-reduction process (2e^−^ ORR) is considered a promising method for economic, decentralized, and safe onsite H_2_O_2_ production without secondary pollution [1,14]. Photoelectrocatalysis could synergistically enhance catalytic performance as compared to photocatalysis and electrocatalysis alone [1]. To date, there are few reports on PEC ORR to H_2_O_2_. In 2016, Jakešová et al. carried out a seminal work employing a hydrogen-bonded organic semiconductor photocathode (the yellow pigment epindolidione@Ag substrate) for PEC ORR towards H_2_O_2_ generation [15]. The performance of the H_2_O_2_ yield exceeded prior reports on photocatalysts by one to two orders of magnitude. Since then, both inorganic semiconductor- and metal-complex-based electrode materials catalyzing oxygen reduction to hydrogen peroxide have received much attention. Mase et al. [16] achieved selective H_2_O_2_ production via 2e^−^ ORR on a cobalt complex (Co^II^(Ch))-modified counter electrode without the need for external bias. Gryszel and co-workers used inorganic heterojunction film photocathodes (NiFeO_x_/BiVO_4_) for the production of high concentrations of H_2_O_2_, with the Faradaic efficiency remaining at around 70% [17]. Zhang et al. [18] discovered that a BiVO_4_ photoanode with an electrodeposited layer of SnO_2_ almost completely prevented oxygen evolution reaction (OER) competition and H_2_O_2_ decomposition by hole reoxidation, finally achieving a high H_2_O_2_ yield (86%). Choi et al. [19] constructed a PEC cell using a ruthenium-decorated TiO_2_ photoelectrode, which is able to continuously produce hydrogen peroxide over 100 h with a concentration of ~80 mM. Ma and colleagues [20] demonstrated Gd^3+^-doped CuBi_2_O_4_/CuO heterojunction film photocathodes that favored the 2e^−^ ORR pathway for selective production of H_2_O_2_ by modifying the electronic structure of the CuBi_2_O_4_ electrode surface. Compared with the pristine CuBi_2_O_4_/CuO photocathode, Gd^3+^ doping led to a six-fold increase in hydrogen peroxide concentration, reaching 1.3 mM within 30 min. NiO [21] and CuBi_2_O_4_/CuO [22] photocathodes also exhibited efficient ORR activity for hydrogen peroxide production.

In addition to inorganic photocathode materials, organic, metal complex, and/or their composite/hybrid photocathodes, which are mainly deposited by drop coating and electropolymerization, have received increasing attention. Wamser et al. [23] investigated electropolymerized films of porphyrin polymers (pTAPP) and its cobalt complex pCoTAPP on glassy carbon, and demonstrated the electrochemical and photoelectrochemical production of H_2_O_2_ via a two-electron reduction of oxygen with good Faradaic efficiency and modest TON values. Photoelectrochemically, pCoTAPP exhibited a low overpotential for ORR to hydrogen peroxide. Li et al. investigated a metal-free poly-terthiophene (pTTh) photocathode for efficiently producing a higher concentration of H_2_O_2_ (110 mM) in an alkaline solution [24]. They found that the ORR was dependent on the pH of electrolytes; the NiFeO_x_/BiVO_4_–pTTh dual-photoelectrodes in PEC devices achieved bias-free synthesis of H_2_O_2_ for several cycles without any noticeable decay. Theoretical calculations showed that the selectivity-determining step in the 2e^−^ pathway is over ~200 times faster than that in the 4e^−^ process. Zhang et al. showed that the positively surface-charged carbon dot could be used as an electrolyte for PEC oxygen reduction to H_2_O_2_ [25]. Despite significant progress in this area, much work is required for PEC ORR performance improvement by developing metal-coordination polymer-based materials and deriving structure-activity relationships.

Recently, we have reported on the first example of an oxidative electropolymerized film based on a vinyl-containing homoleptic Ru(II) complex [26]; the oxidative eletropolymerization overcame the following disadvantages of the conventional reductive electropolymerization previously reported for the vinyl groups on Ru(II) complexes [27]: a relatively fast scan rate, strict deoxygenation of electrolyte solution, and relatively low electrochemical activity and poor adhesion of the resulting electropolymerized films [28,29]. To verify the general applicability of the oxidative electropolymerization strategy for the vinyl group on Ru(II) complexes, we successfully extended the oxidative electropolymerization strategy to a vinyl-containing heteroleptic Ru complex [Ru(**L**)_2_(bpy)](PF_6_)_2_ **Ru1** (where **L** is 1-phenyl-2-(4-vinylphenyl)-1*H*-imidazo[4,5-*f*][1,10]phenanthroline, bpy is 2,2′-bipyridine) to yield a porous electropolymerized film, poly**Ru1**. To the best of our knowledge, poly**Ru1** is the first Ru-complex-based electropolymerized film electrode that can efficiently perform both solar photoelectric conversion/photocurrent polarity switch and photoelectrosynthesis of H_2_O_2_ in a membraneless cell. Herein, we are reporting on these interesting findings.

## 2. Results and Discussions

### 2.1. Synthesis and Electropolymerization

**Scheme 1:**
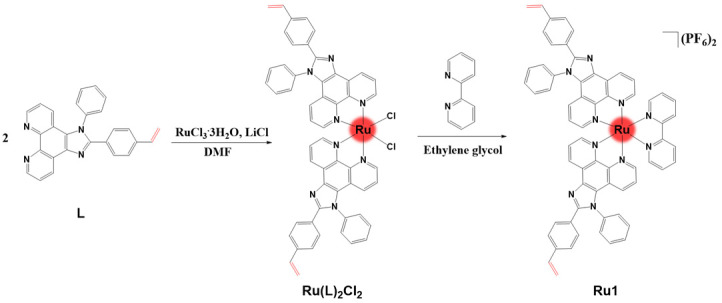
Synthetic route to **Ru1**.

Ru(**L**)_2_Cl_2_ and **Ru1** were synthesized according to Scheme 1 and characterized by ^1^H NMR, ion trap mass spectrometry and elemental analysis. The nuclear magnetic resonance signals at about 6.74, 5.92 and 5.34 ppm are attributed to the H atoms on the vinyl group in complexes Ru(**L**)_2_Cl_2_ (Figure S1) and **Ru1** (Figure S2). The total number of protons are 36 and 44 for Ru(**L**)_2_Cl_2_ and **Ru1**, respectively, which are compatible with the theoretical ones. The observed *m*/*z* value of 527.75 in the mass spectrum of **Ru1** (Figure S3) is in good agreement with the calculated *m*/*z* value of 527.14 for [**Ru1**-2PF_6_^−^]^2+^.

**Scheme 2:**
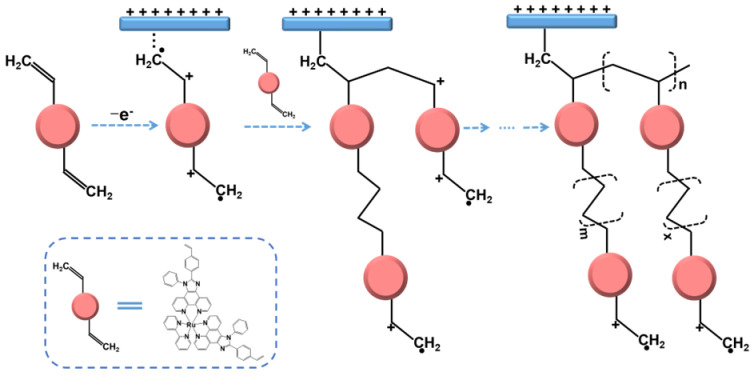
The proposed electropolymerization mechanism of **Ru1**^2+^.

The electropolymerization of **Ru1** was carried out using the potentiodynamic technique through repetitive cyclic voltametric scans. **Ru1** displayed two oxidation peaks at approximately +1.44 and +1.28 V vs. Ag (Figure S4). Both the anodic/cathodic peak currents increased, and the anodic peak potentials shifted towards more positive potentials with increasing scan cycle numbers, indicating the successive deposition of a conductive polymer film onto the ITO surface, and an increase in the degree of polymerization, which was accompanied by an increase in film thickness. By employing this oxidative electropolymerization method, we prepared varying thicknesses of the electropolymerized films on the ITO electrode by adjusting the number *n* of potential scan cycles, poly(**Ru1**)*_n_* (*n* = 3, 5, 8, 10 and 13). With reference to earlier reports [26,30,31], we found that both styryl- containing organic small molecules and metal complexes can form thin films through anodic electropolymerization. Therefore, we propose a mechanism for the oxidative electropolymerization of **Ru1** as shown in Scheme 2. The styrene portion produced a highly active radical-carbonyl cation intermediate under initial electroinitiation and was quickly adsorbed/grafted to the electrode surface. The adsorbed/grafted radical-carbonyl cationic intermediates then attacked other monomers containing the styrene groups, forming polymers containing sp^3^ hybridized C-C bonds.

### 2.2. UV–Vis Absorption and Emission Spectroscopy

**Figure 1:**
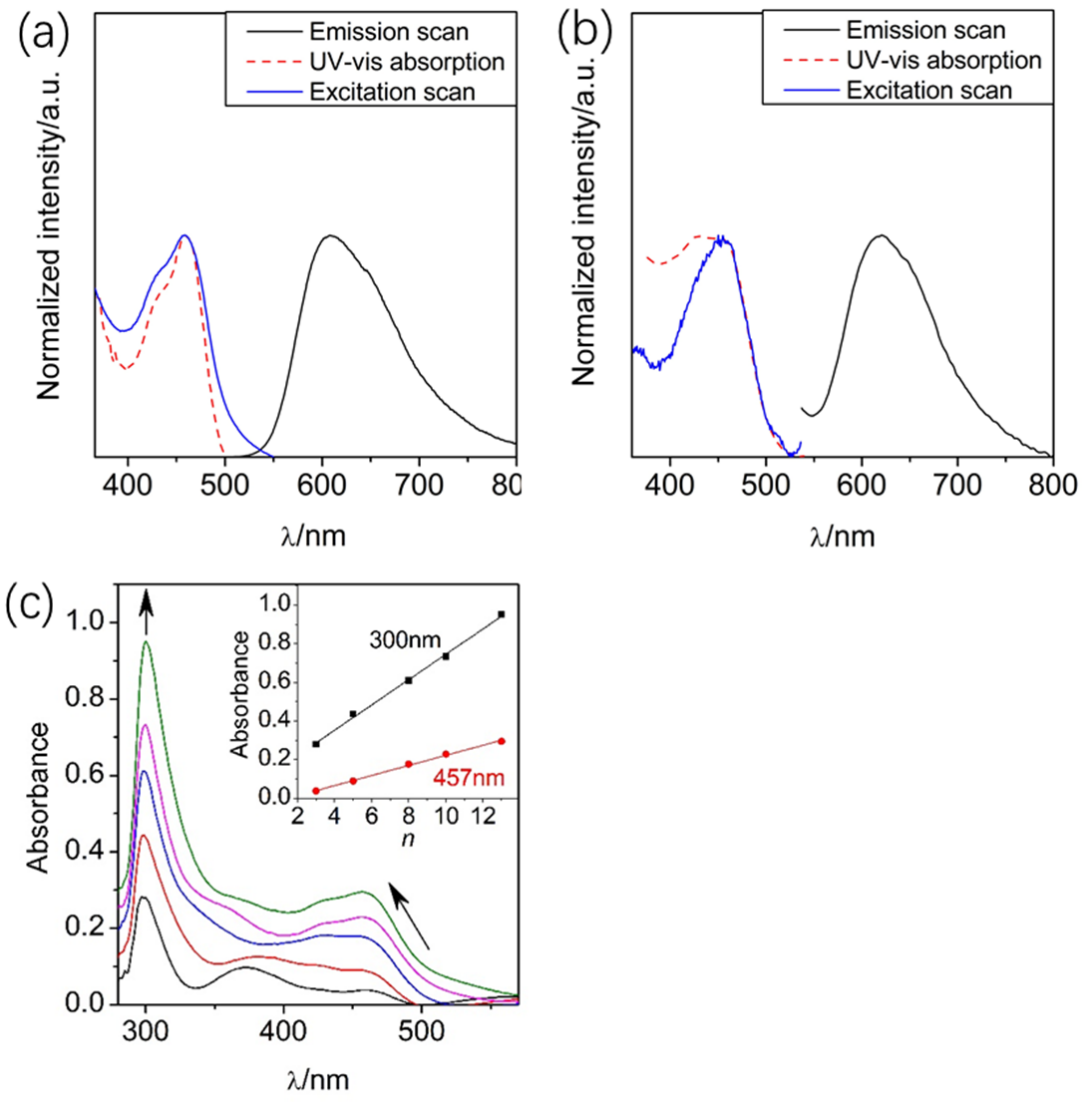
(**a**) UV–Vis absorption, excitation and emission spectra of **Ru1** in acetonitrile (*λ*_ex_/*λ*_em_ = 459 nm/608 nm, *c* = 5 × 10^−6^ M); (**b**) UV–Vis absorption, excitation and emission spectra of poly(**Ru1**)_8_@ITO (*λ*_ex_/*λ*_em_ = 456 nm/620 nm); and (**c**) UV–Vis absorption spectra of films poly(**Ru1**)*_n_* (*n* = 3, 5, 8, 10, 13)@ITO. The inset shows the linear relationship between the absorbances at 300 and 457 nm and the number of scan cycles *n*.

UV–Vis absorption, emission and excitation spectra of **Ru1** and poly(**Ru1**)_8_ on ITO are shown in Figure 1. The maximum emission peak of **Ru1**, which can be attributed to triplet metal-to-ligand charge transfer (^3^MLCT) emission, is located at 608 nm, a 12-nm blue shift from the emission peak of poly(**Ru1**)_8_ at 620 nm. The absorption spectra of **Ru1** and poly(**Ru1**)_8_ are consistent with their excitation spectra (Figure 1a,b), indicating that the observed emission originated from the excitation of **Ru1** and poly(**Ru1**)_8_ rather than an impurity. The UV–Vis spectra for poly(**Ru1**)*_n_* (*n* = 3, 5, 8, 10, 13) on the ITO electrodes are displayed in Figure 1c. They have three characteristic absorption bands that are centered at 300, 370, and 457 nm, and their absorption intensities at these wavelengths increased linearly with the potential scan cycle numbers, proving that the equal amount of poly(**Ru1**) was deposited on ITO at each potential scan cycle. The surface concentration *Γ* (mol·cm^−2^) of **Ru1** in the polymer films was determined according to Equation (1), a modified version of the Beer−Lambert law for two-dimensional film [32]:

*Γ* = 10^−3^ *D*/*ε* (1)

where *D* is the absorbance of poly(**Ru1**)*_n_* films at 457 nm, calculated as 0.0261 according to the slope in the linear relationship in Figure 1c inset; *ε* is the molar absorption coefficient (M^−1^·cm^−1^) of the **Ru1** moiety in the polymer films, which was approximated by the ε value of **Ru1** in acetonitrile at 457 nm (1.92 × 10^4^ M^−1^·cm^−1^). The surface concentration *Γ* of poly(**Ru1**)*_n_* films was determined to be 1.36 × 10^−9^ mol/cm^2^, which is similar to the *Γ* values [(1.33~1.98) × 10^−9^ mol/cm^2^] of analogous Ru(II) complexes in their electropolymerized films reported previously [26,33,34].

### 2.3. X-Ray Photoelectron Spectroscopy (XPS)

The chemical compositions of monomer **Ru1** powder and film poly(**Ru1**)_8_@ITO were characterized by XPS. The binding energies for each peak were calibrated with the C_1s_ peak at 284.6 eV. High-resolution XPS spectra of **Ru1** powder (Figure S5a) and poly(**Ru1**)_8_@ITO (Figure S5d) show that the electron-binding energies of Ru3d_5/2_ are almost the same, with values of 280.7 and 280.8 eV, respectively. Moreover, the N1s peaks for the **Ru1** powder were found to be located at 398.2 and 399.8 eV, which are almost the same as those (398.3 and 399.9 eV) for the film (Figure S5b,e). The electron-binding energies of Ru3d_5/2_ and N1s observed in the electropolymerization film and those in the precursor **Ru1** reveal little difference, which is consistent with the electropolymerization mechanism, as presented in Scheme 2. The full-range XPS spectra of both the **Ru1** powder (Figure S5c) and poly(**Ru1**)_8_@ITO (Figure S5f) exhibited strong Ru3d, C1s and N1s peaks at binding energies of ~281, ~285 and ~400 eV, respectively. The appearance of the energy peaks at ~136 eV for P2p and 685 eV for F1s indicate that PF_6_^−^ existed in the **Ru1** powder and poly(**Ru1**)_8_ film as the counter anions of the **Ru1** complex cations. The O1s peak was also found at 532.7 eV, which originated from the ITO substrate [35].

### 2.4. Scanning Electron Microscopy (SEM)

**Figure 2:**
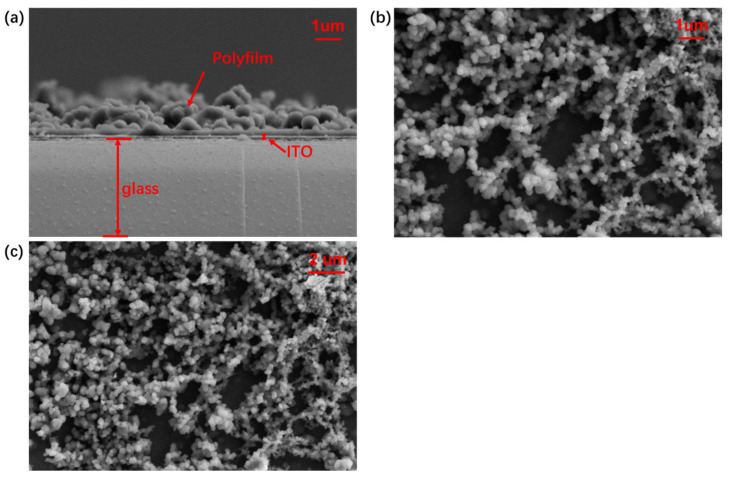
Side-view (**a**) and top-view (**b**,**c**) SEM images of poly(**Ru1**)_20_@ITO.

The surface morphology and cross-sectional view of poly(**Ru1**)_20_@ITO were investigated by SEM (Figure 2). As shown in Figure 2a, the poly(**Ru1**)_20_ film with a thickness of about 2 μm was successfully assembled onto the ITO surface. That is to say, a thickness of around 100 nm was achieved for each potential scanning cycle, which is much thicker than the thickness we previously reported on for the electropolymerized film of a triphenylamine-containing Ru complex [33]. As seen in Figure 2b, the poly**Ru1** film possesses an inhomogeneous and porous surface structure.

### 2.5. Electrochemical Studies

**Figure 3:**
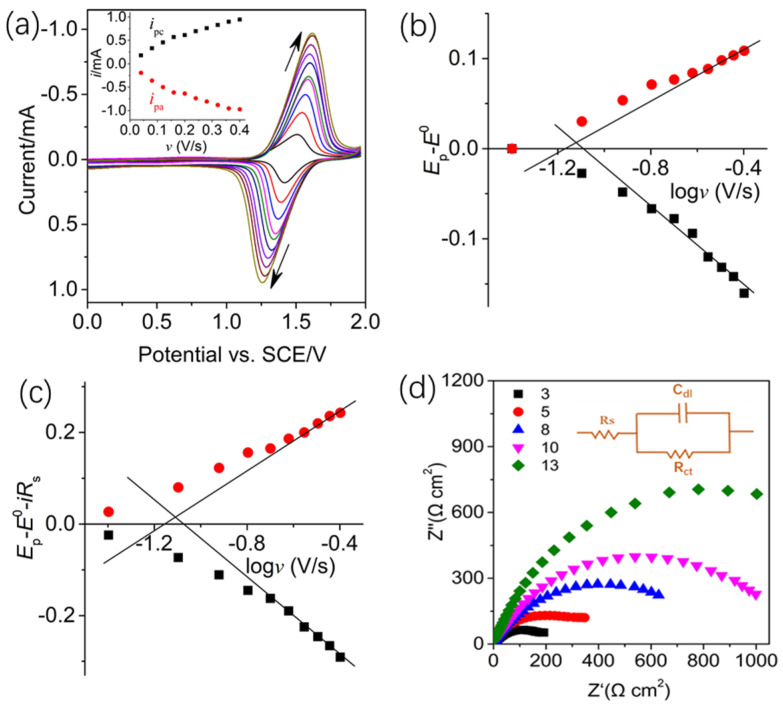
(**a**) Cyclic voltammograms of the poly(**Ru1**)_3_ films in CH_2_Cl_2_ solution that were recorded upon increasing the scan rate (ν) from 0.04 to 0.4 V/s—the inset shows linear dependence of the peak current on ν. The dependence of the anodic (circle) and cathodic (square) overpotentials without (**b**) and with (**c**) corrections of ohmic drop on the logarithm of the potential scan rate (log ν); and (**d**) nyquist diagrams obtained in 0.1 M Na_2_SO_4_ and 5 mM [Fe(CN)_6_]^3−/4−^ electrolyte solution at 0.123 V in the range from 100 kHz to 1 Hz for poly(**Ru1**)*_n_* (*n* = 3, 5, 8, 10, 13) films.

Figure 3 shows the cyclic voltammograms (CVs) of poly(**Ru1**)_3_@ITO in the **Ru1** monomer-free 0.1 M Bu_4_NPF_6_/CH_2_Cl_2_ solution recorded at different potential scan rates from 0.04 to 0.4 V/s. Each CV displays one reversible redox couple at an *E*_1/2_ value of +1.47 V vs. SCE, which is the same *E*_1/2_ value we previously reported on for an electropolymerized poly(**Ru2**)_1_ film [26], attributable to the Ru^III/II^ redox process. The slow interfacial electron transfer rate could not keep up with the potential scanning rate, which caused the potential separation between the cathodic and anodic waves to increase from 108 mV at 0.04 V/s to 160 mV at 0.4 V/s. A linear dependence of the anodic and cathodic peak currents was observed at scan rates between 0.04 and 0.4 V/s (Figure 3a inset), indicating the surface-controlled redox processes in the well-adhered polymer film. This behavior conforms to the adsorbed redox species in a thin-layer cell, according to Equation (2):

*i*_p_ = [(*n*F)^2^*AΓv*]/(4*RT*) (2)

The peak current (*i*_p_), number of electrons involved in the electrode reaction (*n*), Faraday’s constant (*F*) with a value of 96,485 C/mol, electrode area (*A*) of 3 cm^2^, surface concentration in mol·cm^−2^ (*Γ*), and potential scan rate in V/s represent the variables in this equation. The calculated surface concentration of the ruthenium-based redox center on the ITO electrode was found to be 1.13 × 10^−9^ mol·cm^−2^, which is slightly lower than the *Γ* value determined by UV–Vis spectroscopy, indicating of the presence of highly electroactive species in the poly(**Ru1**)_3_ film. Furthermore, the *Γ* value of the poly(**Ru1**)_3_ film is 2.6-fold higher than that of poly(**Ru2**)_1_ film derived from cyclic voltammetry [26]. The heterogeneous electron transfer rate constant (*k*_s_) was derived by analyzing nonlinear plots (Figure 3b) depicting *E*_p_ − *E*^o^ vs. the log*v*, in which *E*_p_ refers to the anodic peak potential (*E*_pa_) or cathodic peak potential (*E*_pc_) and *E*^o^ represents the average of *E*_pa_ and *E*_pc_ at the slow scan rate. According to the Equations (3) and (4) of Laviron’s theory [36], the *k*_s_ value could be determined with α representing electron transfer coefficient while *v*_a_ and *v*_c_ are critical scan rates obtained through straight line fitting for anodic and cathodic data shown in Figure 3b respectively; their values were found to be approximately equal to 0.06960 and 0.08156 respectively.

(3) $$\frac{\alpha }{1-\alpha }=\frac{v_{\alpha }}{v_{c}}$$

(4) $$k_{s}=\frac{nF\alpha v_{c}}{RT}=\frac{nF(1-\alpha )v_{\alpha }}{RT}$$

A *k*_s_ value for the poly(**Ru1**)_3_ film was derived to be 1.46 s^−1^, which is larger than the values of 0.33 s^−1^ and 0.91 s^−1^ we previously reported for the TPA-based electropolymerized film poly(**Ru3**)_1_ [33] and the thiophene-based electropolymerized film poly(**Ru4**)_1_ [34], respectively. As anticipated, the thicker films of poly(**Ru1**) (*n* = 5, 8, 10 and 13) exhibited lower apparent *k*_s_ values (Figures S6–S9, Table S1). The *k*_s_ value was also derived by nonlinear plotting *E*_p_ − *E*^o^ − *iR*_s_ instead of *E*_p_ − *E*^o^ vs. the log*v* (Figure 3c), taking into account the ohmic drop of the solution. Here, *R*_s_ and the *i* value represent the solution resistance and the oxidation or the reduction peak current at each scan rate [37], respectively. By using an *R*_s_ value of 138.2 Ω cm^2^ obtained by electrochemical impedance spectroscopy, a corrected *k*_s_ value for poly(**Ru1**)_3_ film was calculated as 1.50 s^−1^, which is higher than the corrected *k*_s_ value of 0.51 s^−1^ for poly(**Ru3**)_1_ [33] and slightly lower than the corrected *k*_s_ value of 1.65 s^−1^ for poly(**Ru4**)_1_ [34]. The above facts indicate that the vinyl-appended poly(**Ru1**)_3_ film exhibited a moderately fast electron transfer rate compared to poly(**Ru3**)_1_ and poly(**Ru4**)_1_.

The electron transfer behavior of electropolymerized films was investigated using electrochemical impedance spectroscopy (EIS) and permeability experiments using [Fe(CN)_6_]^3−/4−^ as redox probes. The EIS measurements were performed in a 5 mM [Fe(CN)_6_]^3−/4−^ solution containing 0.1 M Na_2_SO_4_ as a supporting electrolyte to obtain electrochemical impedance spectra for poly(**Ru1**)*_n_* (*n* = 3, 5, 8, 10, 13) (Figure 3d). In these spectra, the linear part in the low-frequency range of the Nyquist diagrams represents the diffusion control, while the semicircle part in the high-frequency range refers to the charge transfer kinetics of probe [Fe(CN)_6_]^3−/4−^. The EIS parameters (Table S2) obtained by fitting the data according to the equivalent electrical circuit (the inset of Figure 3d) based on the Roberts and Crowell model [38], provided insights into the film properties. Increases in film thickness led to significant increases in *R*_ct_ values from 229.2 to 2015 Ω cm^2^, indicating further restriction on the movement of the probe [Fe(CN)_6_]^3−/4−^ within thicker films. This behavior is consistent with the film-thickness dependence of the apparent *k*_s_ values. By comparing the CVs of redox probe of [Fe(CN)_6_]^3−/4−^ obtained utilizing poly(**Ru1**)*_n_* (*n* = 3, 5, 8, 10, 13) films and blank ITO as the working electrodes, the permeability of poly(**Ru1**)*_n_* films was investigated, as shown in Figure S10. The peak current value of the probe [Fe(CN)_6_]^3−/4−^ decreased rapidly with increasing *n* up to at least *n* = 13, indicating that the permeability of the thin film became worse with the increase in film thickness. This result is consistent with the presence of smaller and fewer holes in the thicker films, as revealed by the SEM. The above-mentioned results could be rationalized by the fact that the diffusion of probe molecules [Fe(CN)_6_]^3−/4−^ into electropolymerized film became difficult due to steric hindrance as the films became thicker.

The electrochemical stability of surface-bound poly(**Ru1**)_8_ film was examined by repeated CV cycles in 0.1 M Bu_4_NPF_6_/CH_2_Cl_2_ solution. After 50 potential sweeping cycles, minimal loss of the Ru^III/II^-based redox wave was observed (Figure S11). The decrease in the peak current was less than 34.7%, indicating that the film had good adhesion and electrochemical stability.

### 2.6. Photocurrent Generation Behaviors

**Figure 4:**
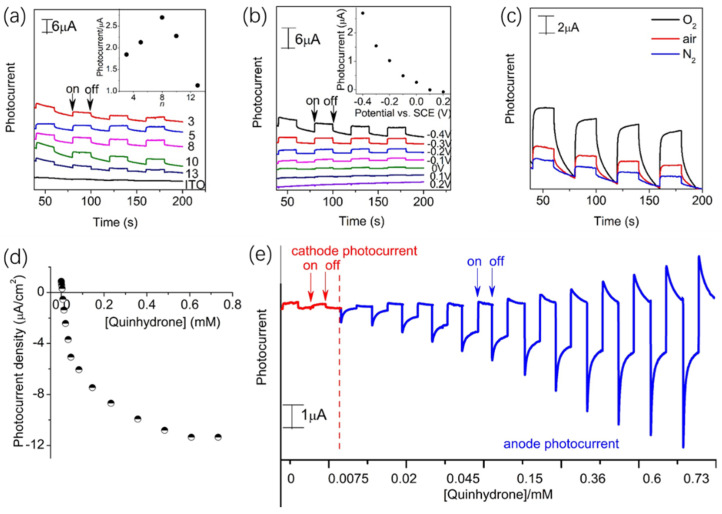
(**a**) Photocurrent responses for bare ITO and poly(**Ru1**)*_n_* (*n* = 3, 5, 8, 10, 13) films biased at −0.4 V vs. SCE—the inset shows a plot of photocurrent vs. potential scan cycles number *n*; (**b**) photocurrent responses for poly(**Ru1**)_8_ film biased at −0.4, −0.3, −0.2, −0.1, 0, +0.1 and 0.2 V vs. SCE—the inset shows a plot of photocurrent vs. potential; (**c**) photocurrent responses for poly(**Ru1**)_8_ film in N_2_−, air−, and O_2_−equilibrated electrolyte solutions biased at −0.4 V vs. SCE; (**d**) effect of quinhydrone on photocurrent density of poly(**Ru1**)_8_ biased at 0 V vs. SCE in 0.1 M Na_2_SO_4_ aqueous solution; and (**e**) photocurrent switching behavior induced by changes in quinhydrone concentrations.

As shown in Figure 4a, as compared to the blank ITO, the electropolymerized films poly(**Ru1**)*_n_* (*n* = 3, 5, 8, 10, 13) generated stable and obvious cathodic photocurrents that were film-thickness-dependent. The photocurrent decreased with increases in film thickness, which was probably due to the fact that the increased interfacial charge transfer resistance and recombination of the generated carriers offset the increases in the light-harvesting capacity of the film [32]. We further explored the effect of bias potentials on the photocurrent of poly(**Ru1**)_8_ film (Figure 4b). The photocurrents continued to increase at more negative potentials, indicating that the interfacial electron transfer occurred from ITO electrode to the poly(**Ru1**)_8_ film and to the electrolyte solution, generating the cathodic photocurrents. It is worth mentioning that among poly(**Ru1**)*_n_* (*n* = 3, 5, 8, 10, 13) films, the poly(**Ru1**)_8_ film exhibited a maximum photocurrent (photocurrent density) of 2.7 μA (9.64 μA/cm^2^), which compares favorably with some representative electrostatic self-assembly films and other electropolymerized films, as listed in Table S3. At a zero-bias potential, a moderate cathode photocurrent (photocurrent density) of 0.26 μA (0.93 μA/cm^2^) was observed for the poly(**Ru1**)_8_ film.

Usually, the photocurrent is strongly influenced by electron acceptors or donors in the electrolyte solution, which can affect the directional electron transfer between the film-modified ITO and electrolyte solution. O_2_ present in the electrolyte solution could act as an electron acceptor, leading to an increase in cathodic photocurrent. Compared with the N_2_-saturated electrolyte solution, the photocurrent increased by 2.4 times, reaching 6.0 μA in the O_2_−saturated electrolyte solution (Figure 4c). On the contrary, increasing concentrations of an electron donor, such as quinhydrone, in the electrolyte solution resulted in a gradual decrease in cathodic photocurrents (Figure 4d), as quinhydrone hindered electron flow from ITO to the electrolyte solution. Interestingly, when the quinhydrone concentration reached 0.0075 mM, a switching in photocurrent polarity from cathodic to anodic photocurrents was observed. As can be interestingly seen from Figure 4e, a maximum anodic photocurrent (photocurrent density) of 3.18 μA (11.4 μA/cm^2^) was achieved at a quinhydrone concentration of 0.73 mM, which is greater than those for previously reported analogous electropolymerized films of poly**Ru2** film (2.58 μA or 9.22 μA/cm^2^) [26] and poly(Bdb)_1_−modified ITO {Bdb = 4,4′-[[2,2′-bipyridine]-5,5′-diyldi(1E)-2,1-ethenediyl]bis[*N*,*N*-diphenyl-benzenamine} (0.98 μA or 3.5 μA/cm^2^) [39].

**Figure 5:**
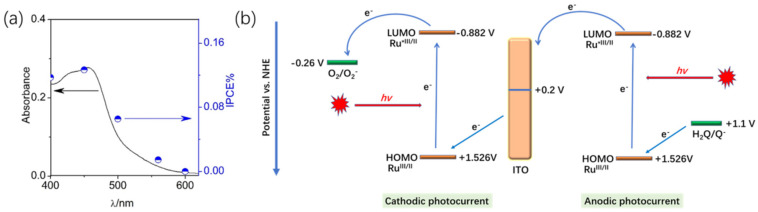
(**a**) Comparison of photocurrent action spectrum (scattered) and UV–Vis absorption spectrum (solid line) of poly(**Ru1**)_8_ film at zaro applied potential vs. SCE in 0.1 M Na_2_SO_4_ aqueous solution containing 0.73 mM quinhydrone; and (**b**) the proposed mechanism of cathodic and anodic photocurrent generation.

As shown in Figure 5a, the photocurrent action spectrum and UV–Vis absorption spectrum in the range of 400~620 nm of poly(**Ru1**)_8_@ITO are almost identical, indicating that the photocurrent was generated by the photoexcitation of poly(**Ru1**)_8_. According to Equation (5) [40], the photon-to-current conversion efficiency (IPCE) value at 450 nm was calculated as 0.127%, which is moderate compared to those for previously reported electropolymerized films (see Table S3).

(5) $$IPCE(\%)=\frac{1240J_{\mathrm{sc}}}{\lambda \phi_{m}}\times 100$$

where $\phi_{m}$, λ, and *J*_sc_ represent the flux density of incident monochromatic light (mW·cm^−2^), the wavelength of incident light (nm), and the short−circuit photocurrent density of poly(**Ru1**)_8_ film (mA·cm^−2^), respectively.

Figure 5b shows the mechanism for the generation of anodic and cathodic photocurrents in the electropolymerized films. The energy values of the highest-occupied molecular orbital (HOMO) for poly**Ru1** were derived to be 1.526 V vs. NHE using the onset oxidation potential in the cyclic voltammetry [41]. Additionally, the value of the lowest unoccupied molecular orbital (LUMO) was calculated to be −0.882 V vs. NHE according to Equations (6) and (7), utilizing an onset absorption wavelength (λ_onset_ in nm) value of 515 nm for poly**Ru1**.

(6) $$E^{00}=\frac{1240}{\lambda_{\mathrm{onset}}}$$

*E*_LUMO_ = *E*_HOMO_ + *E*^00^ (7)

The energy level of the ITO electrode’s conduction band and the reduction potential of O_2_ and the oxidation potential of H_2_Q are taken to be +0.2 V (4.70 eV below the vacuum level) [42], and −0.26 V [43] and +0.11 V vs. NHE [43], respectively. The electron flow responsible for the cathode photocurrent is depicted on the left side of Figure 5b, which can be described as follows: under light illumination, electron transition occurred from the HOMO to the LUMO of poly(**Ru1**), followed by electron transfer to electron acceptor O_2_ molecules in the electrolyte solution; Subsequently, electrons from the ITO conduction band flow towards the HOMO orbital of poly**Ru1**. In contrast, as shown on the right side of Figure 5b, the anodic photocurrent was caused by the following reversed electron flow: upon light excitation, electrons moved from the HOMO to the LUMO within poly**Ru1** before transferring to the ITO conduction band; oxidized poly**Ru1** was then regenerated through gaining electrons from donor molecules in the electrolyte solution (quinhydrone); conversely, oxidized quinhydrone acquired electrons from the counter electrode to complete the anodic photocurrent cycle.

### 2.7. Photoelectrocatalytic Oxygen Reduction to Hydrogen Peroxide

**Figure 6:**
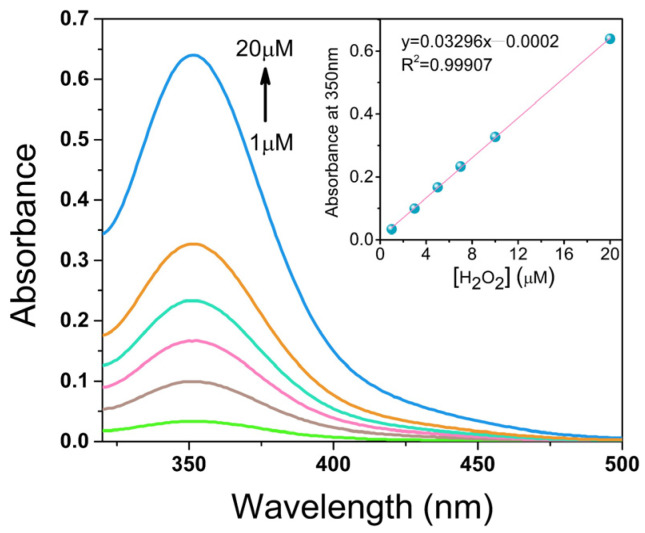
UV–Visible absorption spectra of iodide−molybdate indicator solution containing varying concentrations of H_2_O_2_. Inset shows a linear fitting of the absorbances at 350 nm vs. H_2_O_2_ concentrations.

**Figure 7:**
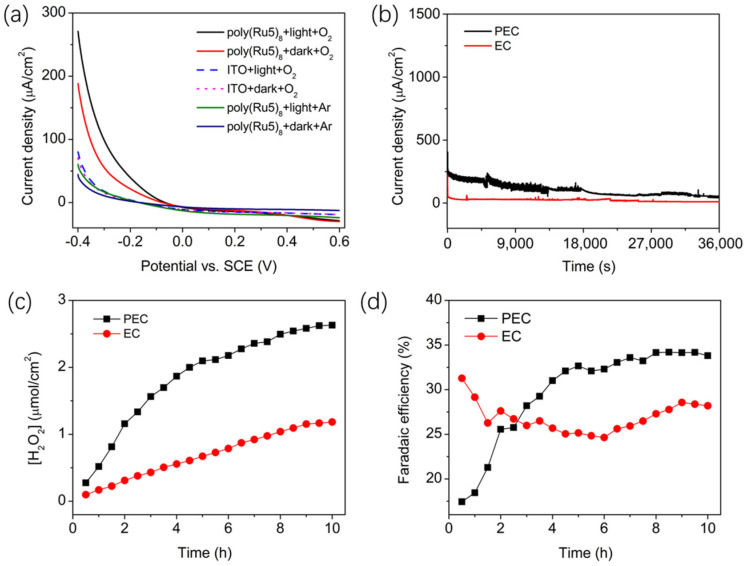
(**a**) Cyclic voltammograms of poly(**Ru1**)_8_ film and blank ITO in O_2_− and Ar− equilibrated 0.1 M Na_2_SO_4_ aqueous solution at pH 7.0 with and without 60 mWcm^−2^ of white light illumination; (**b**) the corresponding *i*−t curve; (**c**) H_2_O_2_ production during the photoelectrocatalytic process; and (**d**) changes in H_2_O_2_ production Faradaic efficiencies for poly(**Ru1**)_8_ film over 10 h photoelectrocatalysis under 60 mWcm^−2^ white light irradiation at a biased potential of −0.4 V vs. SCE in O_2_-equilibrated 0.1 M Na_2_SO_4_ aqueous solution at a pH of 7.0.

To evaluate the amount of H_2_O_2_ generated and the Faradaic efficiency (FE) for the electropolymerized film poly(**Ru1**)_8_@ITO, the catalyst film was tested in a Teflon cell containing 0.1 M Na_2_SO_4_ electrolyte aqueous solution at a pH of 7.0. H_2_O_2_ was quantitatively determined by means of the spectrophotometric molybdenum-triiodide method [44]. As shown in Figure 6, the standard curve was a linear fitting of the solution absorbances vs. H_2_O_2_ concentrations, and an extinction coefficient (*ε*) of I_3_^−^ at 350 nm was derived to be 3.30 × 10^4^ M^−1^ cm^−1^, which is consistent with the literature values of 3.12 × 10^4^ [44] and 2.64 × 10^4^ M^−1^cm^−1^ [45]. The cyclic voltammograms of the poly(**Ru1**)_8_ film and blank ITO in the O_2_- and Ar-equilibrated 0.1 M Na_2_SO_4_ aqueous solution are presented in Figure 7a. When exposed to light or darkness, the poly(**Ru1**)_8_ film immersed in the O_2_-equilibrated electrolyte delivered an obvious increase in current density, in contrast to the lack of appreciable rises in the current density observed in the Ar-equilibrated electrolyte solution. As anticipated, no obvious difference in current density was observed for the blank ITO electrode when immersed into O_2_- or Ar-equilibrated electrolyte solution under illuminated and dark conditions. Such results indicated that the poly(**Ru1**)_8_ film showed superior ORR photoelectrocatalytic activity. The onset PEC and EC ORR potential values at a pH of 7 were shown to be 0.54 V and 0.52 V vs. SCE, respectively [17]. In addition, we further explored the effects of film thickness and applying bias potentials on the production of H_2_O_2_, FE, and the turnover number (TON). We found that the optimal film thickness for H_2_O_2_ generation was *n* = 8, which attained the greatest H_2_O_2_ production of 0.277 μmol/cm^2^ with a TON of 175 after 0.5 h of white light illumination when biased at −0.4 V vs. SCE (Figure S12). When the bias potentials became more negative from −0.1 V to −0.4 V, the production of H_2_O_2_ and FE became higher, indicating that the optimal bias voltage was −0.4 V (Figure S13). As can be seen from the photocurrent–time curves in Figure 7b, prolonged testing of the poly(**Ru1**)_8_ film photocathode exhibited stable photocurrents over 10 h of photoelectrocatalytic and electrocatalytic processes under illumination and without illumination, respectively, indicating that the photoelectrocatalysis/electrocatalysis we report on here have outstanding long-time durability for ORR in a neutral electrolyte aqueous solution. As shown in Figure 7c, the concentrations of H_2_O_2_ gradually increased with time and reached 1.18 μmol/cm^2^ in the dark after continuous 10 h electrocatalysis at a bias potential of −0.4 V vs. SCE. After continuous 10 h photoelectrocatalysis, the poly(**Ru1**)_8_ film under illumination showed an approximately 1.3-time increase in H_2_O_2_ production (2.63 μmol/cm^2^). In other words, the poly(**Ru1**)_8_ film has enhanced PEC ORR activity and stability compared to that of the EC ORR. Faradaic efficiency (FE) was calculated by integrating the current passed during a photoelectrocatalysis experiment using chronoamperometry, which equals the experimentally observed H_2_O_2_ molecular numbers divided by the theoretical H_2_O_2_ molecular numbers [23]. By counting the number of moles of electrons traveled and applying a molar ratio of two electrons per mole of H_2_O_2_, one may determine the theoretical amount of H_2_O_2_. The FE in the dark was well maintained at 28% even after 10 h electrocatalysis, implying that the poly(**Ru1**)_8_ film exhibited excellent electrocatalytic stability for H_2_O_2_ generation (Figure 7d). Under light illumination, the H_2_O_2_ photoelectrocatalysis generation FE of poly(**Ru1**)_8_ film increased by 34%. It is worth noting that compared with the recently reported two-electron ORR photocathods (Table S4), such as pTAPP and pCoTAPP [23], the TON and H_2_O_2_ production of the ITO@poly(**Ru1**)_8_ electrode were outstanding.

**Figure 8:**
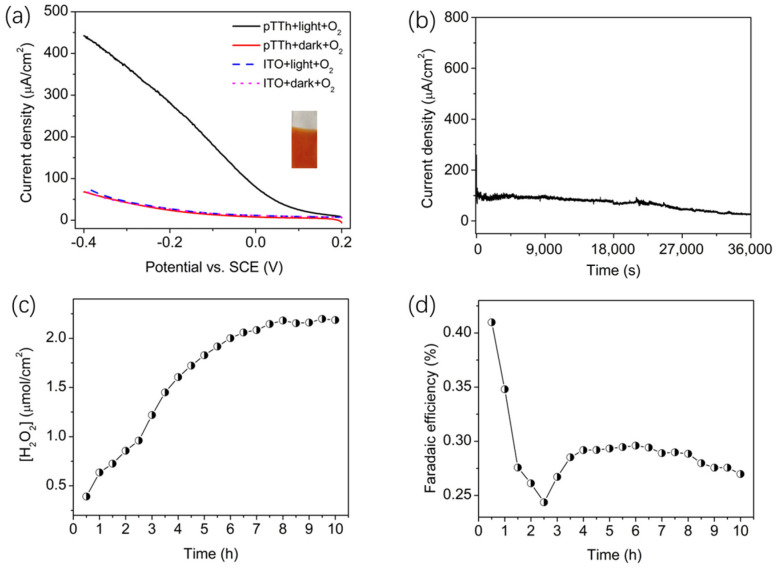
Cyclic voltammograms (**a**) of pTTh film and blank ITO in O_2_−equilibrated 0.1 M Na_2_SO_4_ aqueous solution at pH 7.0 with and without 60 mWcm^−2^ of white light illumination; photocurrent–time curve (**b**); changes in H_2_O_2_ concentrations (**c**); and Faradaic efficiencies (**d**) vs. duration of photoelectrocatalysis of PECs based on pTTh photocathode in O_2_-equilibrated 0.1 M Na_2_SO_4_ aqueous solution (pH 7.0) when the photocathode was biased at a potential of −0.4 V vs. SCE and irradiated with 60 mWcm^−2^ of white light.

In order to comparatively study the photoelectrochemical properties, we prepared the polyterthiophene (pTTh) film onto ITO according to the literature [24] and tested the PEC ORR performance under the same experimental conditions as described for poly(**Ru1**) films. The color of the pTTh@ITO film matched that of pTTh@CP in the previous literature reports. As shown in Figure 8a, a higher photocurrent density was obtained for pTTh@ITO under illumination compared to darkness, indicating superior PEC activity to the EC one. The durable photoelectrocatalysis of pTTh was carried out via the chronoamperometric method at a fixed potential of −0.4 V vs. SCE (Figure 8b), which showed that 72% of the original current was maintained after a 6 h electrolysis at a stable current value of ~50 μA cm^−2^. Figure 8c displays the time courses of H_2_O_2_ production at the photocathodic side. The yield of hydrogen peroxide increased to 2.18 μmol·cm^−2^ and remained unchanged after 8 h photocatalysis. To be specific, the amount of hydrogen peroxide produced by the thin film pTTh is much lower than its literature value, mainly due to the single-compartment cell used in this paper. As shown in Figure 8d, the initial FE was close to 41%, and then decayed to around 29%. Therefore, under the identical experimental conditions described herein, the poly(**Ru1**)_8_@ITO exhibited a better PEC ORR performance for hydrogen peroxide generation than the pTTh.

## 3. Materials and Methods

### 3.1. Materials and Common Instrumentation

**L** was synthesized according to a previously reported method [26]. All other precursor reagents and solvents were purchased from commercial sources and used as received without further purification. Indium-tin oxide (ITO)-coated glass substrates with a sheet resistance of 10 Ω·cm^2^ were obtained from commercial sources and cleaned as before [26]. 1H NMR spectra were recorded on a Bruker (Billerica, MA, USA) ARX-400 spectrometer. The ion trap mass spectrum was analyzed on AB SCIEX. Elemental analyses were carried out on a Vario (El Cajon, CA, USA) EL elemental analyzer. Luminescence and UV–Vis absorption spectra were captured using a Hitachi (Tokyo, Japan) FS5 spectrophotometer and UV-2450 UV–Vis spectrophotometer, respectively. The surface morphology and thickness of poly**Ru1** films were investigated using scanning electron microscopy (SEM (Tokyo, Japan), Hitachi S-4800-II). X-ray photoelectron spectroscopy was conducted to examine the compositions of these films by Thermo Fisher (Waltham, MA, USA) Hitachi S-4800-II.

### 3.2. Synthesis of Ru(**L**)_2_Cl_2_

A suspension of **L** (159.2 mg, 0.4 mmol), RuCl_3_·3H_2_O (52.2 mg, 0.2 mmol), and LiCl (169.6 mg, 4 mmol) in *N*,*N*-dimethylformamide (6 mL) was heated at 150 °C for 12 h under N_2_ protection before cooling to room temperature. A mixed solvent of 10 mL acetone and 10 mL water was added to the reaction solution and left it in a refrigerator overnight. A purplish black solid was collected by filtration, washed with water, and then vacuum-dried to provide 60 mg of product in a 31% yield. ^1^H NMR (δ_H_, ppm, 400 MHz, *d*_6_-DMSO): 9.16 (dd, *J*_1_ = 12.4 Hz, *J*_2_ = 8.0 Hz, 1 H), 8.28 (s, 1 H), 7.94~7.80 (m, 8 H), 7.71 (t, *J* = 5.2 Hz, 8 H), 7.65~7.60 (m, 6 H), 7.52~7.48 (m, 6 H), 6.74 (dd, *J*_1_ = 17.6 Hz, *J*_2_ = 11.2 Hz, 2 H), 5.92 (d, *J* = 17.6 Hz, 2 H), 5.34 (d, *J* = 11.2 Hz, 2 H). Analysis calculated for C_54_H_36_Cl_2_N_8_Ru·5.7H_2_O: C, 60.53; H, 4.46; N, 10.46; found: C, 60.57; H, 4.31; N, 10.28.

### 3.3. Synthesis of [Ru(**L**)_2_(bpy)](PF_6_)_2_ (bpy = 2,2′-bipyridine) **Ru1**

A suspension of bpy (14 mg, 0.09 mmol) and Ru(**L**)_2_Cl_2_ (85 mg, 0.09 mmol) in ethylene glycol (10 mL) was heated at 150 °C for 12 h under N_2_ protection before cooling to room temperature. The precipitate, formed after a saturated aqueous NH_4_PF_6_ solution was added to the reaction solution, was collected by filtration and was purified by column chromatography on silica gel using CH_2_Cl_2_/C_2_H_5_OH (*v*/*v*, 5:1) as an eluant. Recrystallization from acetonitrile/ether after most of the solvent was driven off by rotary evaporation, afforded 35 mg of a deep-red product in a 35.3% yield. ^1^H NMR (δ_H_, ppm, 400 MHz, *d*_6_-DMSO): 9.18 (m, 2 H), 8.92 (m, 2 H), 8.19~8.01 (m, 4 H), 7.98 (d, *J* = 5.2 Hz, 2 H), 7.79~7.66 (m, 16 H), 7.57 (t, 4 H), 7.51~7.39 (m, 8 H), 6.72 (dd, *J*_1_ = 18.4 Hz, *J*_2_ = 10.8 Hz, 2 H), 5.90 (d, *J* = 17.6 Hz, 2 H), 5.33 (d, *J* = 10.8 Hz, 2 H). Ion trap MS (in CH_3_CN): calculated, *m*/*z* = 527.14 ([M − 2PF_6_^−^]^2+^); found, *m*/*z* = 527.75. Analysis calculated for C_64_H_44_F_12_N_10_P_2_Ru·2H_2_O·CH_2_Cl_2_: C, 53.29; H, 3.44; N, 9.56; found: C, 53.29; H, 3.20; N, 9.67.

### 3.4. Electrochemical and Photoelectrochemical Measurements

A CHI-660D electrochemical workstation was used to perform all electrochemical experiments with a 0.1 M Bu_4_NPF_6_ CH_2_Cl_2_ solution as a supporting electrolyte, a silver wire as a pseudo-reference electrode that was calibrated using ferrocene as an internal standard (*E*° = +0.425 V vs. saturated calomel electrode (SCE) [46]), a film-modified ITO glass as the working electrode, and a platinum disk (d = 2 mm) as the counter electrode. The photoelectrochemical measurements were carried out on the CHI-660D electrochemical analyzer with a following three-electrode cell with 0.1 M Na_2_SO_4_ aqueous solution as supporting electrolyte: a film-modified ITO working electrode with an effective area of 0.28 cm^2^, a platinum disk counter electrode (d = 2 mm), and an SCE reference electrode. The polychromatic light irradiation (730 > λ > 325 nm) for the photoelectrochemical measurements was obtained by filtering infrared light through an infrared cutoff filter from a 500 W xenon lamp light source system (Changtuo Co., Ltd., Beijing, China); the monochromatic light for the photocurrent action spectrum measurement was obtained with additional band-pass filters. Light intensity at each wavelength was measured with a ST-900 M photometer (Photoelectric Instrument Factory, Beijing Normal University). A one-chamber type Teflon cell equipped with an ITO working electrode modified with a thin film with an effective area of 2.47 cm^2^ was utilized to carry out the photoelectrochemical oxygen reduction to H_2_O_2_.

## 4. Conclusions

In conclusion, we successfully fabricated porous electropolymerized film poly(**Ru1**)*_n_* (*n* = 3, 5, 8, 10, 13) on ITO electrodes by oxidative electropolymerization of a vinyl-containing Ru(II) complex monomer. The obtained films displayed not only interesting EC properties of reversible redox activity, good EC stability, and a moderately fast electron transfer rate, but also the following intriguing PEC properties: a significant cathodic photocurrent density of 9.64 μA·cm^−2^ at −0.4 V bias potential in the atmosphere condition, and the photocurrent polarity switching from cathodic to anodic with increasing concentrations of quinhydrone; a high anodic photocurrent of 11.4 μA·cm^−2^ at zero bias potential was obtained; and the IPCE value for poly(**Ru1**)_8_ film was found to be 0.127% at 450 nm. More importantly, this novel photocathode performed effective photoelectrosynthesis of H_2_O_2_ in neutral an 0.1 M Na_2_SO_4_ aqueous solution. Within a 10 h photoelectrocatalysis, the PEC cell achieved a higher concentration of H_2_O_2_ production than a pTTh-based PEC cell. This work provides insights into designing more effective photocathode materials for photoelectric conversion/switching, and H_2_O_2_ photoelectrosynthesis devices.

## Data Availability

The data presented in this study are available on request from the corresponding author. The compounds that were used in this paper are available from the authors.

## Supplementary Materials

The following supporting information can be downloaded at: https://www.mdpi.com/article/10.3390/molecules29030734/s1. Figure S1: the ^1^H NMR spectrum of Ru(L)_2_Cl_2_. Figure S2: the ^1^H NMR spectrum of **Ru1**. Figure S3: the ion trap MS spectrum of **Ru1**. Figure S4: cyclic voltammograms of 0.4 mM **Ru1** in CH_2_Cl_2_ solution containing 0.1 M Bu_4_NPF_6_, obtained after different potential scan cycles of 3 (a), 5 (b), 8 (c), 10 (d) and 13 (e) at 0.05 V/s on the ITO. Figure S5: full-range XPS spectra of Ru1 powder (a) and poly(**Ru1**)_8_ film (d); highly resolved XPS spectra of **Ru1** powder (b,c) and poly(**Ru1**)_8_ film (e,f). Figure S6: (a) cyclic voltammograms of poly(**Ru1**)_5_ film in CH_2_Cl_2_ solution that were recorded upon increasing the scan rate (ν) from 0.04 to 0.4 V/s; the inset shows linear dependence of the peak current on ν.; the dependence of the anodic (circle) and cathodic (square) overpotentials (b,c) with corrections of ohmic drop on the logarithm of the potential scan rate (log ν). Figure S7: (a) cyclic voltammograms of poly(**Ru1**)_8_ film in CH_2_Cl_2_ solution that were recorded upon increasing the scan rate (ν) from 0.04 to 0.4 V/s; the inset shows linear dependence of the peak current on ν; the dependence of the anodic (circle) and cathodic (square) overpotentials (b) and (c) with corrections of ohmic drop on the logarithm of the potential scan rate (log ν). Figure S8: (a) cyclic voltammograms of poly(**Ru1**)_10_ film in CH_2_Cl_2_ solution that were recorded upon increasing the scan rate (ν) from 0.04 to 0.4 V/s; the inset shows linear dependence of the peak current on ν; the dependence of the anodic (circle) and cathodic (square) overpotentials (b) and (c) with corrections of ohmic drop on the logarithm of the potential scan rate (log ν). Figure S9: (a) cyclic voltammograms of poly(**Ru1**)_13_ film in CH_2_Cl_2_ solution that were recorded upon increasing the scan rate (ν) from 0.04 to 0.4 V/s; the inset shows the linear dependence of the peak current on ν; the dependence of the anodic (circle) and cathodic (square) overpotentials (b) and (c) with corrections of ohmic drop on the logarithm of the potential scan rate (log ν). Figure S10: cyclic voltammograms of blank ITO and poly(**Ru1**)_n_ (*n* = 3, 5, 8, 10, 13) films in 0.1 M HCl and 1 mM [Fe(CN)_6_]^3−/4−^ solution recorded at a potential scan rate of 0.1 V/s. Figure S11: cyclic voltammograms of poly(**Ru1**)_8_ film in 0.1 M TBAPF_6_ CH_2_Cl_2_ solution recorded by 50 repeated potential scan cycles at 0.1 V/s. Figure S12: (a) changes in H_2_O_2_ concentrations, (b) Faradaic efficiencies over 0.5 h, and (c) TON for poly(**Ru1**)_n_ (*n* = 3, 5, 8, 10, 13) photocathodes vs. n when photocathodes in O_2_-equilibrated at a pH of 7.0 in 0.1 M Na_2_SO_4_ aqueous solution were irradiated with 60 mWcm^−2^ white light and biased at −0.4 V vs. SCE. Figure S13: (a) production of H_2_O_2_, and (b) Faradaic efficiencies over 0.5 h for poly(**Ru1**)_8_; film irradiated with 60 mWcm^−2^ white light and biased at applied potentials from −0.4 to −0.1 V vs. SCE in O_2_-equilibrated at a pH of 7.0 in 0.1 M Na_2_SO_4_ aqueous solution. Table S1: the *Γ* and *k*_s_ values for poly(**Ru1**)_n_ films. Table S2: parameters obtained by fitting the data shown in Figure 3d according to the equivalent electrical circuit of the inset of Figure 3d. Table S3: the comparisons of photocurrent density (*J*) and the IPCE values of Ru complex-based films on ITO. Table S4: selected PEC ORR for H_2_O_2_ parameters for organic-film-modified electrodes. Refs. [47,48] are cited in Supplementary Materials.
