# Supplementary material for: Porous Electropolymerized Films of Ruthenium Complex: Photoelectrochemical Properties and Photoelectrocatalytic Synthesis of Hydrogen Peroxide
Source: Molecules. 2024 Feb 5;29(3):734. doi: 10.3390/molecules29030734 (PMC10856344; doi:10.3390/molecules29030734)
Supplement: Supplementary file 1 [file molecules-29-00734-s001.zip › molecules-2826611-supplementary.pdf]

Supporting Information for

Porous Electropolymerized Films of Ruthenium Complex: Photoelectrochemical Properties and Photoelectrocatalytic Synthesis of Hydrogen Peroxide

Hong-Ju Yin [1,2] and Ke-Zhi Wang [1,*]

[1] Beijing Key Laboratory of Energy Conversion and Storage Materials, College of Chemistry, Beijing Normal University, Beijing 100875, China; yinhongju411@163.com

[2] College of Chemistry and Environmental Science, Qujing Normal University, Qujing 655011, China

* Correspondence: kzwang@bnu.edu.cn; Tel.: +86-10-58805476; Fax: +86-10-58802075

Contents:

EXPERIMENTAL SECTION

Figure S1. The ^{1}H NMR spectrum of **$Ru(L)_2Cl_2$**.

Figure S2. The ^{1}H NMR spectrum of **Ru1.**

Figure S3. The Ion trap MS spectrum of **Ru1.**

Figure S4. Cyclic voltammograms of 0.4 mM **Ru1** in CH_2Cl_2 solution containing 0.1 M Bu_4NPF_6, obtained after different potential scan cycles of 3 (a), 5 (b), 8 (c), 10 (d) and 13 (e) at 0.05 V/s on the ITO.

Figure S5. Full-range XPS spectra of **Ru1** powder (a) and poly(**Ru1**)$_8$ film (d); highly resolved XPS spectra of **Ru1** powder (b, c) and poly(**Ru1**)$_8$ film (e, f).

Figure S6. (a) Cyclic voltammograms of poly(**Ru1**)$_5$ film in CH_2Cl_2 solution that were recorded upon increasing the scan rate (ν) from 0.04 to 0.4V/s; the inset shows linear dependence of the peak current on ν. The dependence of the anodic (circle) and cathodic (square) overpotentials (b) and (c) with corrections of ohmic drop on the logarithm of the potential scan rate (log ν).

Figure S7. (a) Cyclic voltammograms of poly(**Ru1**)$_8$ film in CH_2Cl_2 solution that were recorded upon increasing the scan rate (ν) from 0.04 to 0.4V/s; the inset shows linear dependence of the peak current on ν. The dependence of the anodic (circle) and cathodic (square) overpotentials (b) and (c) with corrections of ohmic drop on the logarithm of the potential scan rate (log ν).

Figure S8. (a) Cyclic voltammograms of poly(**Ru1**)$_{10}$ film in CH_2Cl_2 solution that were recorded upon increasing the scan rate (ν) from 0.04 to 0.4V/s; the inset shows linear dependence of the peak current on ν. The dependence of the anodic (circle) and cathodic (square) overpotentials (b) and (c) with corrections of ohmic drop on the logarithm of the potential scan rate (log ν).

Figure S9. (a) Cyclic voltammograms of poly(**Ru1**)$_{13}$ film in CH_2Cl_2 solution that were recorded upon increasing the scan rate (ν) from 0.04 to 0.4V/s; the inset shows linear dependence of the peak current on ν. The dependence of the anodic (circle) and cathodic (square) overpotentials (b) and (c) with corrections of ohmic drop on the logarithm of the potential scan rate (log ν).

Figure S10. Cyclic voltammograms of blank ITO and poly(**Ru1**)$_n$ (n = 3, 5, 8, 10, 13) films in 0.1 M HCl and 1 mM $[Fe(CN)_6]^{3-/4-}$ solution recorded at a potential scan rate of 0.1 V/s.

Figure S11. Cyclic voltammograms of poly(**Ru1**)$_8$ film in 0.1 M $TBAPF_6$ CH_2Cl_2 solution recorded by 50 repeated potential scan cycles at 0.1 V/s.

Figure S12. (a) Changes in H_2O_2 concentrations, (b) Faradaic efficiencies over 0.5 h, and (c) TON for poly(**Ru1**)$_n$ (n = 3, 5, 8, 10, 13) photocathodes vs. n when photocathodes in O_2-equilibrated at pH 7.0 in 0.1 M Na_2SO_4 aqueous solution were irradiated with 60 $mWcm^{-2}$ white light and biased at -0.4 V vs SCE.

Figure S13. (a) Production of H_2O_2, and (b) Faradaic efficiencies over 0.5 h for poly(**Ru1**)$_8$ film irradiated with 60 $mWcm^{-2}$ white light and biased at applied potentials from -0.4 to -0.1 V vs. SCE in O_2-equilibrated at pH 7.0 in 0.1 M Na_2SO_4 aqueous solution.

Table S1. The Γ and k_s values for poly(**Ru1**)$_n$ films

Table S2. Parameters obtained by fitting the data shown in Figure 3d according to the equivalent electrical circuit of the inset of Figure 3d

Table S3. The comparisons of photocurrent density (J) and the IPCE values of Ru complex-based films on ITO

Table S4. Selected PEC ORR for H_2O_2 parameters for organic film modified electrodes

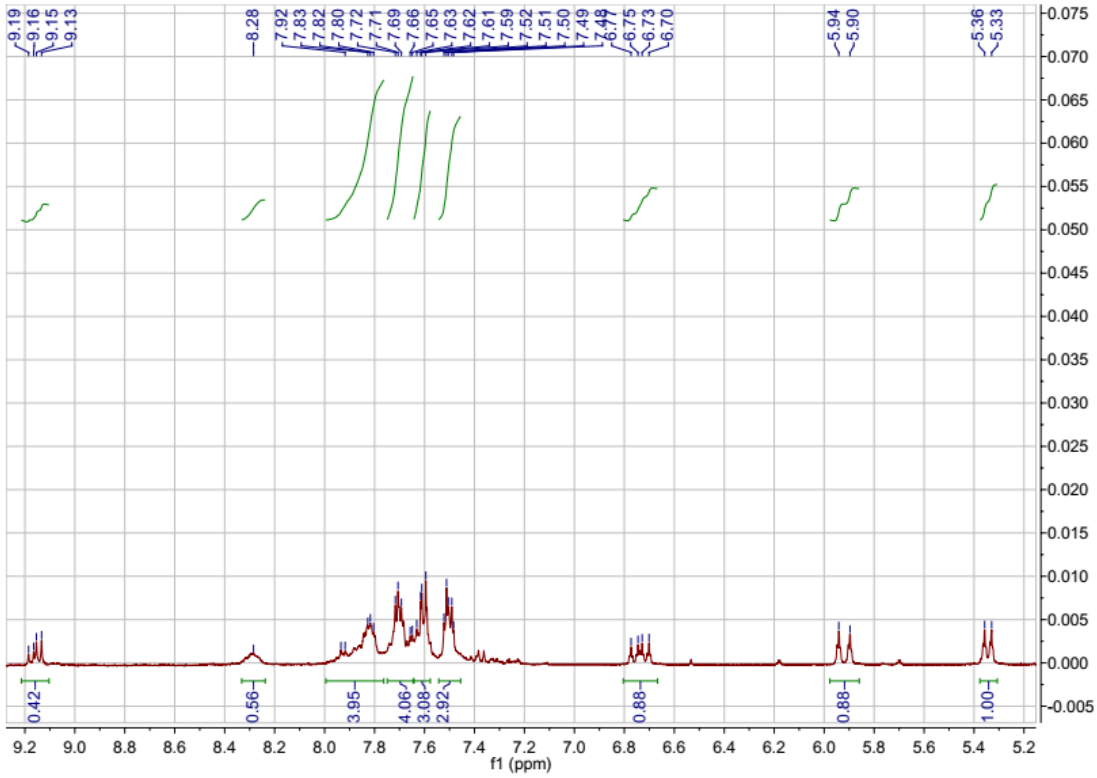

Figure S1. The ^{1}H NMR spectrum of **Ru(L)$_2$Cl$_2$**.

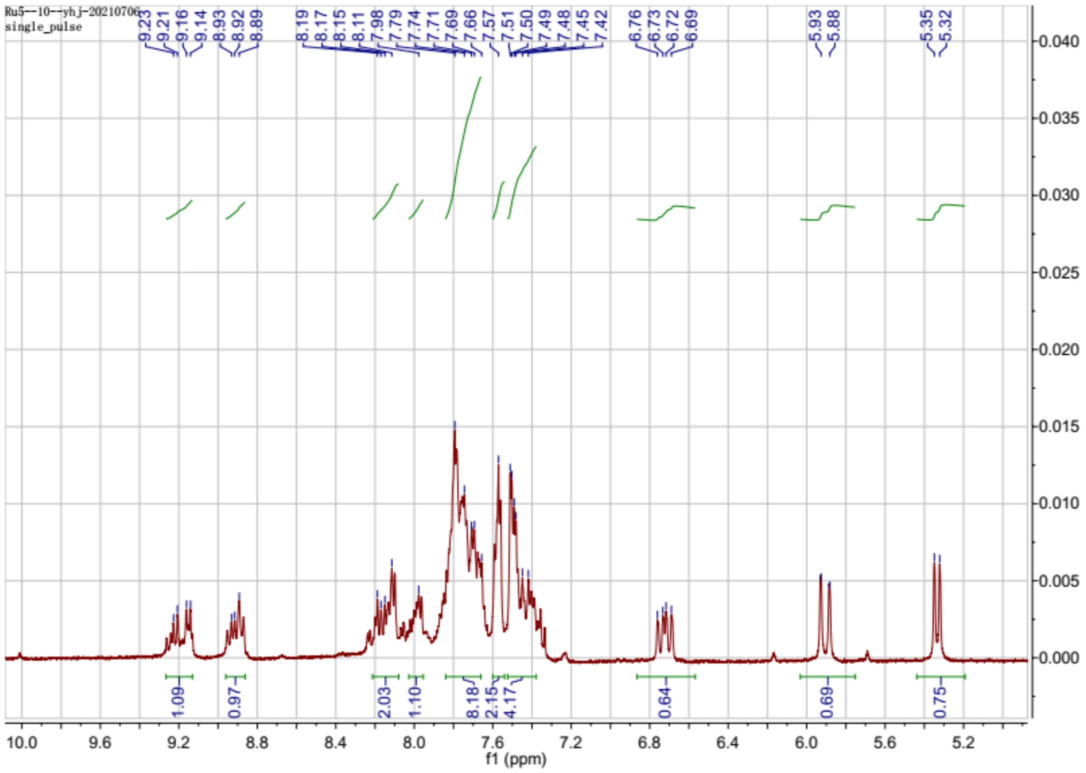

Figure S2. The ^{1}H NMR spectrum of **Ru1.**

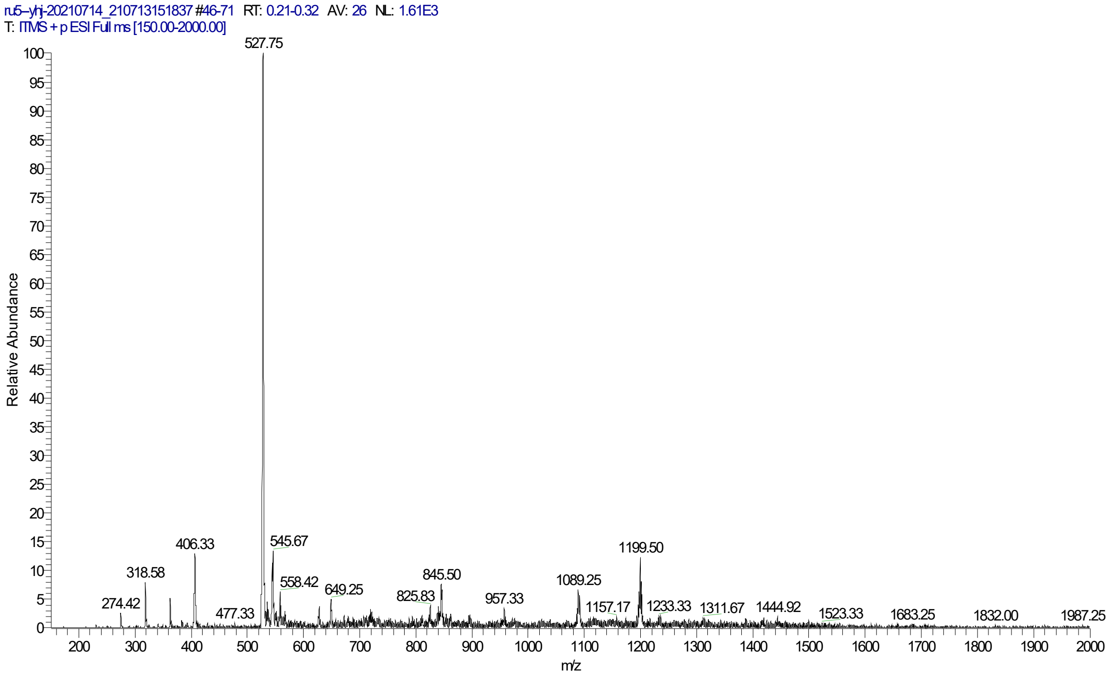

Figure S3. The Ion trap MS spectrum of **Ru1.**

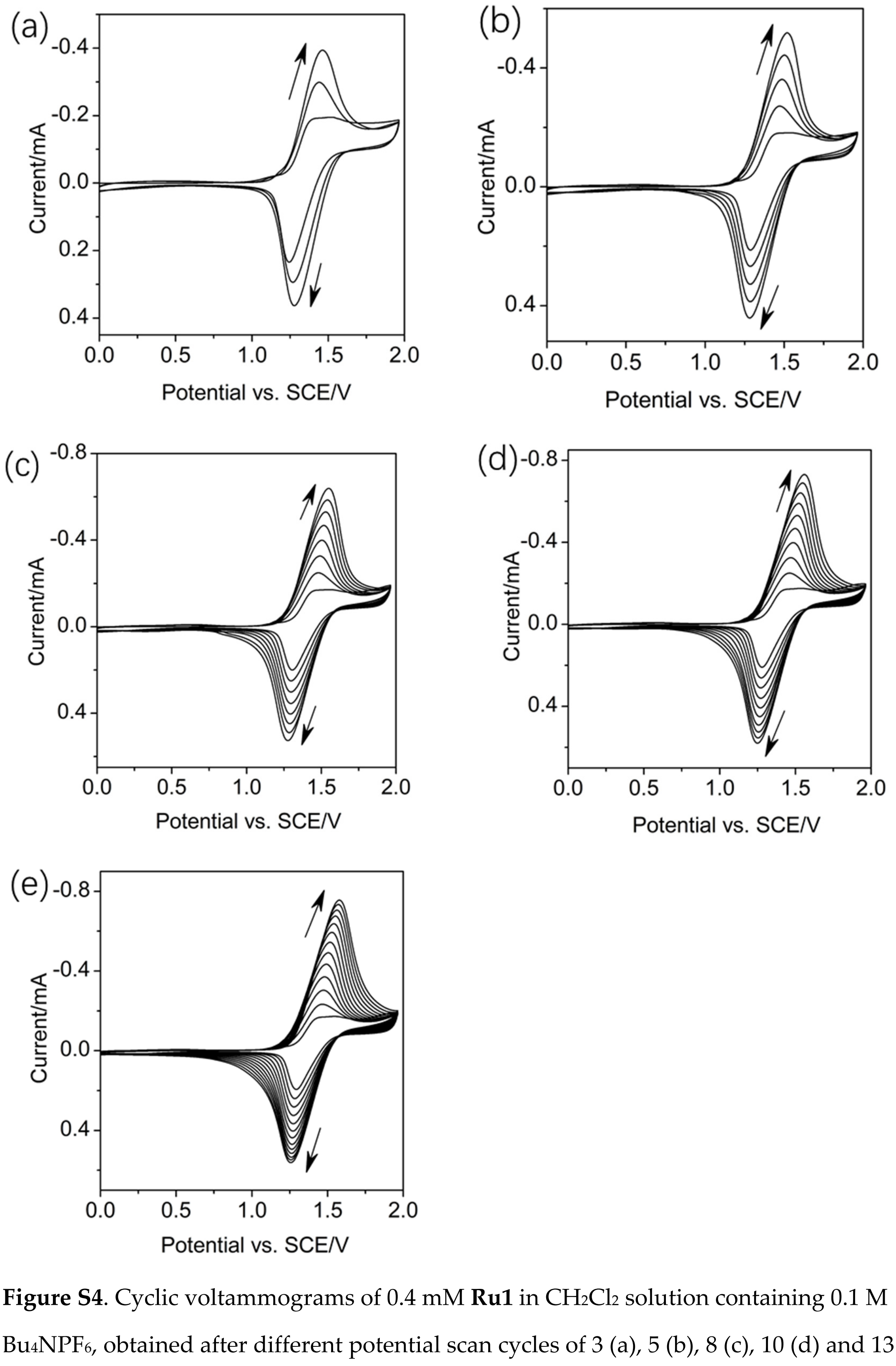

Figure S4. Cyclic voltammograms of 0.4 mM **Ru1** in CH_2Cl_2 solution containing 0.1 M Bu_4NPF_6, obtained after different potential scan cycles of 3 (a), 5 (b), 8 (c), 10 (d) and 13 (e) at 0.05 V/s on the ITO.

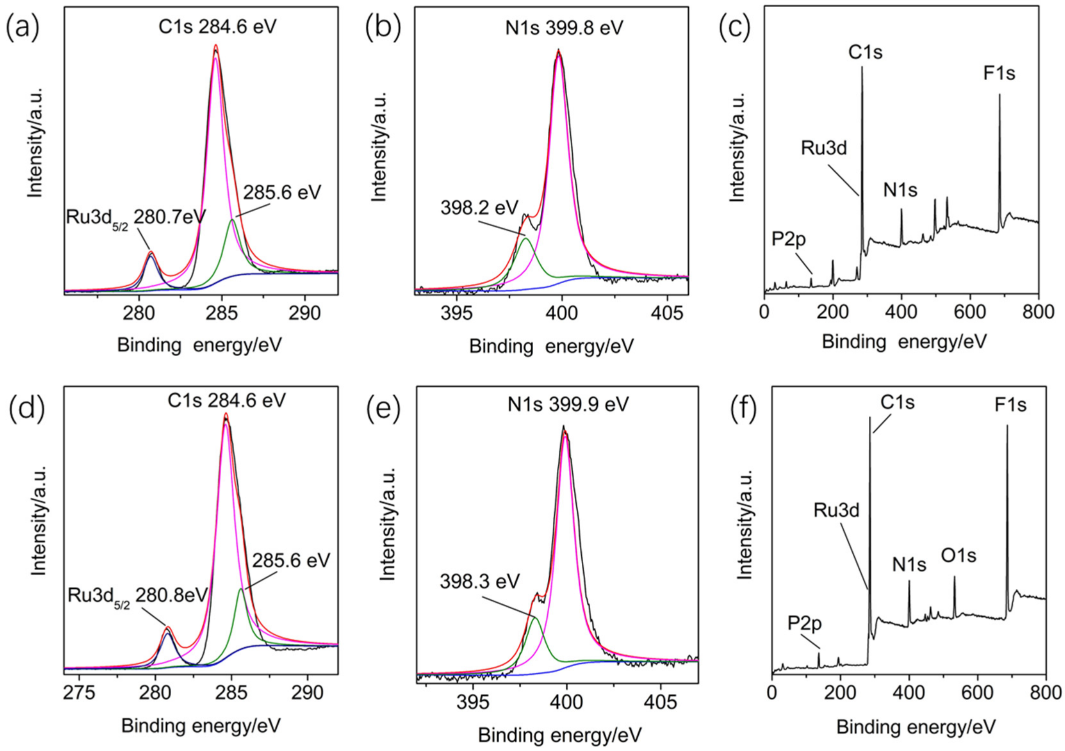

Figure S5. Full-range XPS spectra of **Ru1** powder (a) and poly(**Ru1**)$_8$ film (d); highly resolved XPS spectra of **Ru1** powder (b, c) and poly(**Ru1**)$_8$ film (e, f).

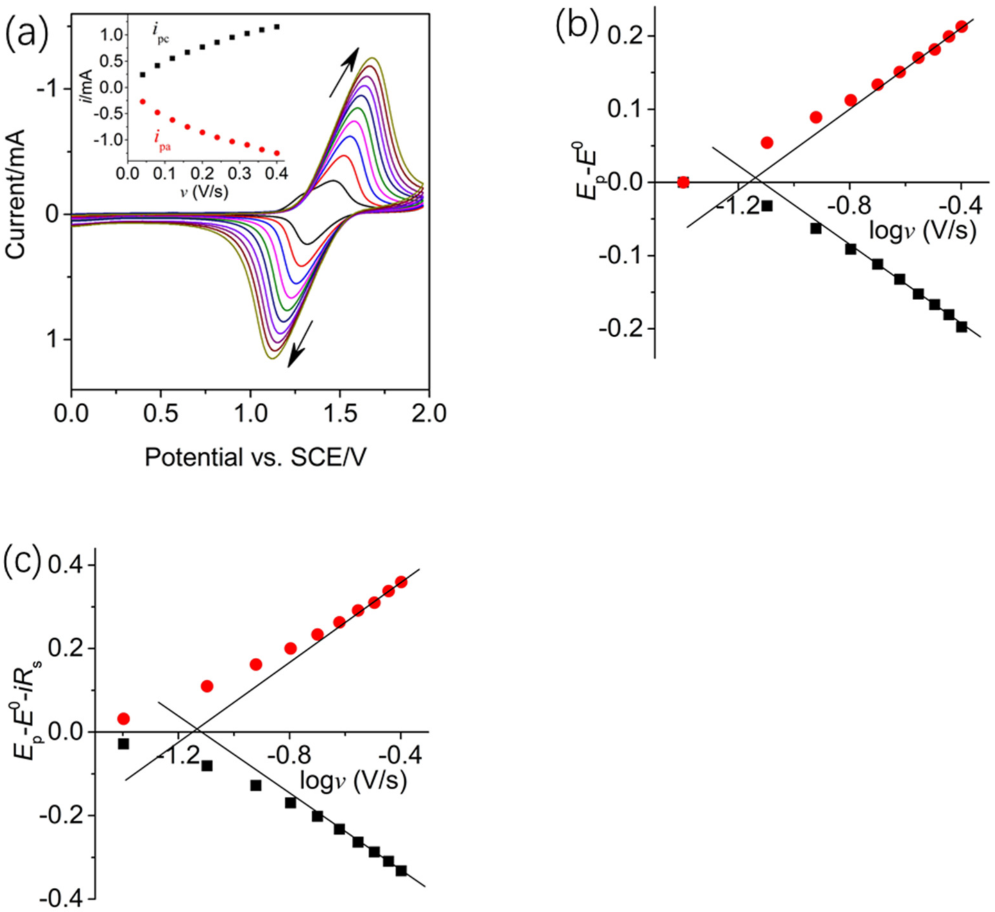

Figure S6. (a) Cyclic voltammograms of poly(**Ru1**)$_5$ film in CH_2Cl_2 solution that were recorded upon increasing the scan rate (ν) from 0.04 to 0.4V/s; the inset shows linear dependence of the peak current on ν. The dependence of the anodic (circle) and cathodic (square) overpotentials (b) and (c) with corrections of ohmic drop on the logarithm of the potential scan rate (log ν).

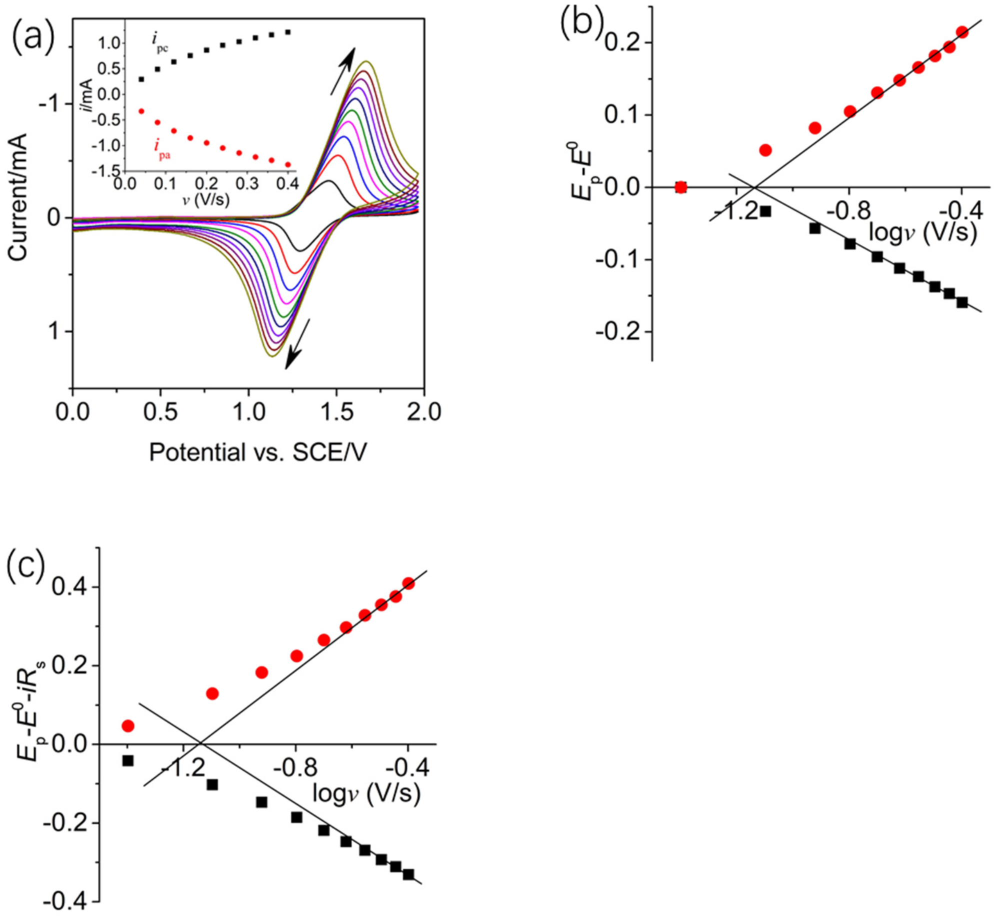

Figure S7. (a) Cyclic voltammograms of poly(**Ru1**)$_8$ film in CH_2Cl_2 solution that were recorded upon increasing the scan rate (ν) from 0.04 to 0.4V/s; the inset shows linear dependence of the peak current on ν. The dependence of the anodic (circle) and cathodic (square) overpotentials (b) and (c) with corrections of ohmic drop on the logarithm of the potential scan rate (log ν).

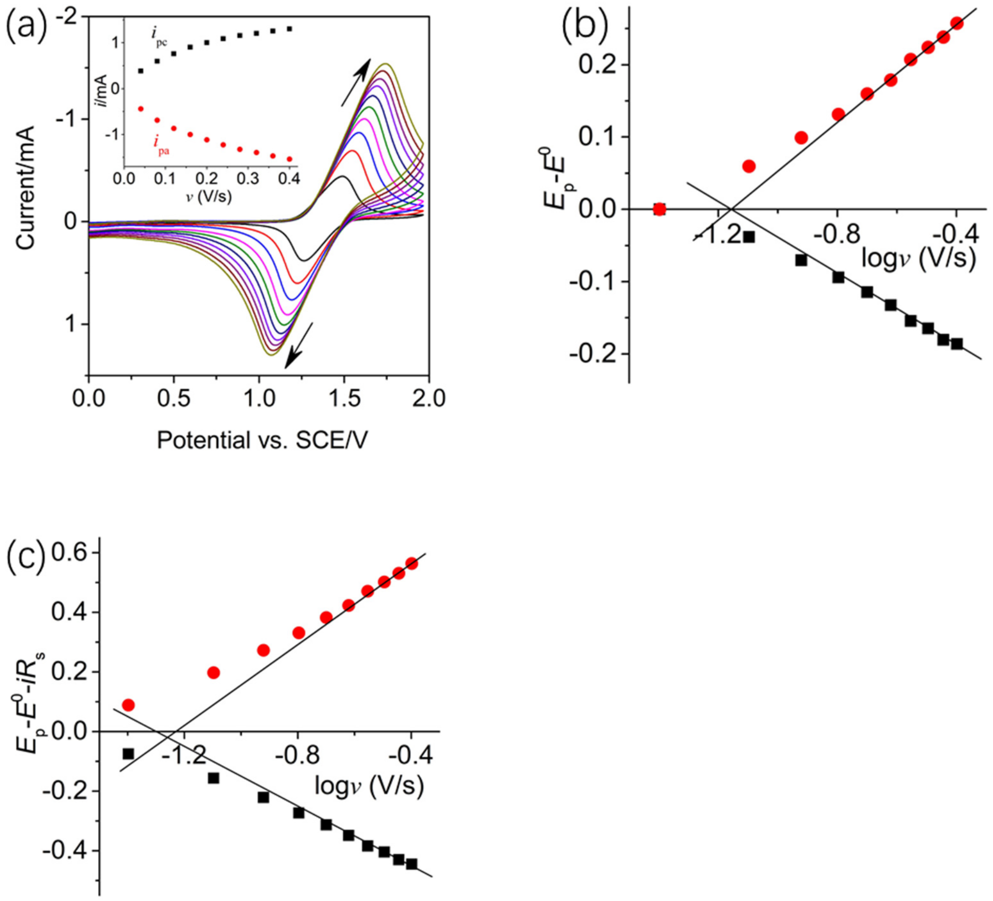

Figure S8. (a) Cyclic voltammograms of poly(**Ru1**)$_{10}$ film in CH_2Cl_2 solution that were recorded upon increasing the scan rate (ν) from 0.04 to 0.4V/s; the inset shows linear dependence of the peak current on ν. The dependence of the anodic (circle) and cathodic (square) overpotentials (b) and (c) with corrections of ohmic drop on the logarithm of the potential scan rate (log ν).

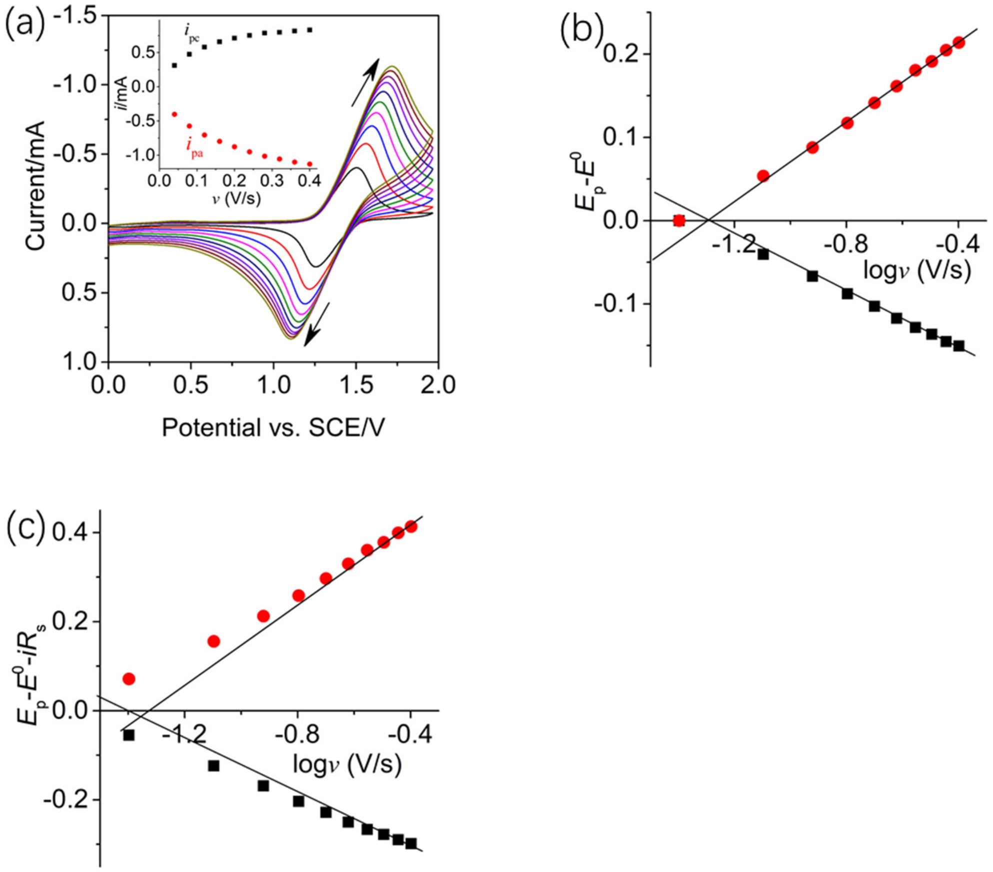

Figure S9. (a) Cyclic voltammograms of poly(**Ru1**)$_{13}$ film in CH_2Cl_2 solution that were recorded upon increasing the scan rate (ν) from 0.04 to 0.4V/s; the inset shows linear dependence of the peak current on ν. The dependence of the anodic (circle) and cathodic (square) overpotentials (b) and (c) with corrections of ohmic drop on the logarithm of the potential scan rate (log ν).

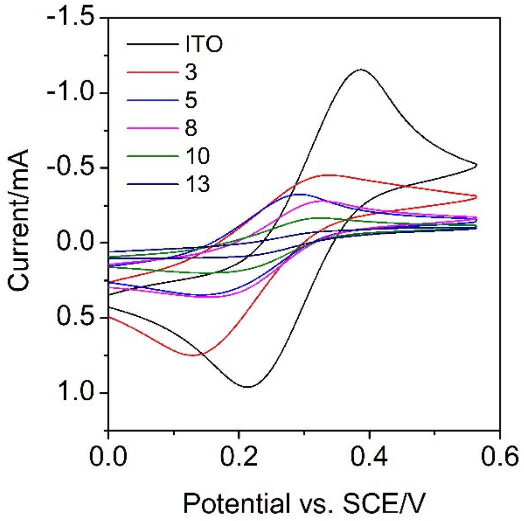

Figure S10. Cyclic voltammograms of blank ITO and poly(**Ru1**)$_n$ (n = 3, 5, 8, 10, 13) films in 0.1 M HCl and 1 mM $[Fe(CN)_6]^{3-/4-}$ solution recorded at a potential scan rate of 0.1 V/s.

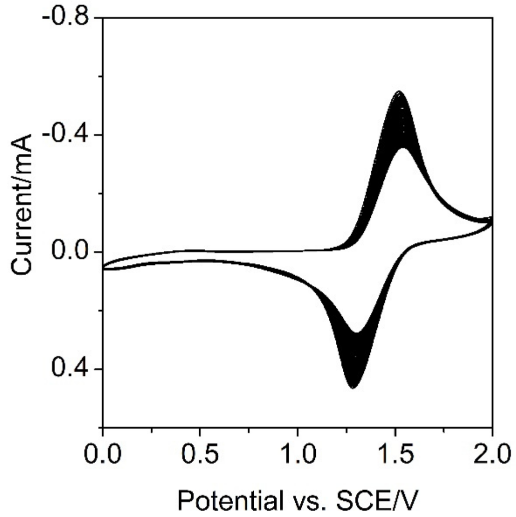

Figure S11. Cyclic voltammograms of poly(**Ru1**)$_8$ film in 0.1 M $TBAPF_6$ CH_2Cl_2 solution recorded by 50 repeated potential scan cycles at 0.1 V/s.

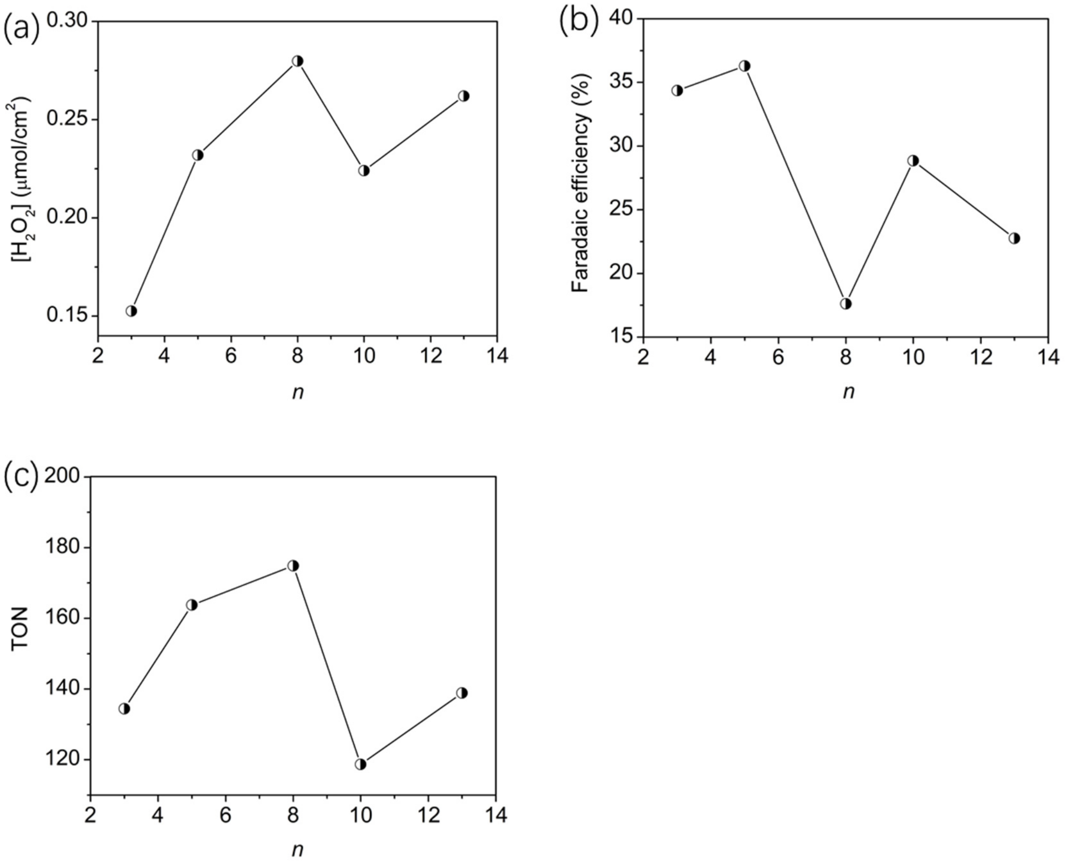

Figure S12. (a) Changes in H_2O_2 concentrations, (b) Faradaic efficiencies over 0.5 h, and (c) TON for poly(**Ru1**)$_n$ (n = 3, 5, 8, 10, 13) photocathodes vs. n when photocathodes in O_2-equilibrated at pH 7.0 in 0.1 M Na_2SO_4 aqueous solution were irradiated with 60 mWcm^{-2} white light and biased at -0.4 V vs SCE.

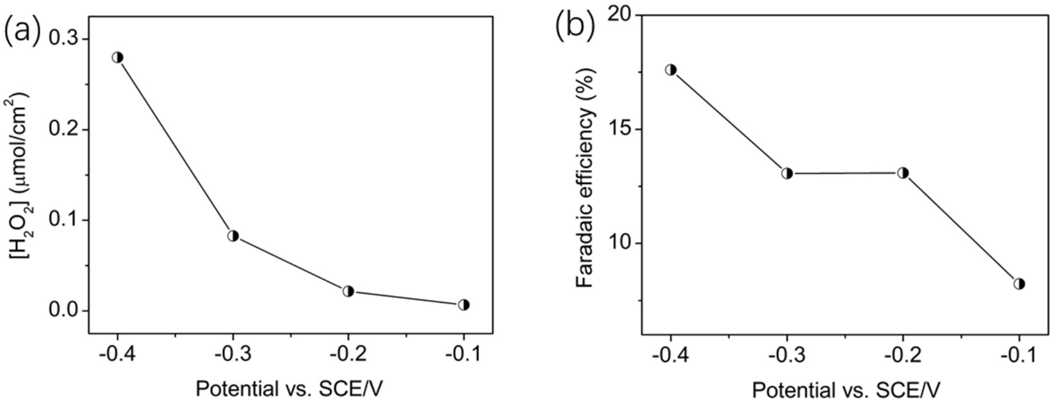

Figure S13. (a) Production of H_2O_2, and (b) Faradaic efficiencies over 0.5 h for poly(**Ru1**)$_8$ film irradiated with 60 mWcm^{-2} white light and biased at applied potentials from -0.4 to -0.1 V vs SCE in O_2-equilibrated at pH 7.0 in 0.1 M Na_2SO_4 aqueous solution.

Table S1. The Γ and k_s values for poly(**Ru1**)$_n$ films

Poly(**Ru1**)$_n$	ν_a **(V/s)**	ν_a **(V/s)**	α	k_s **(s^{-1})**	Γ **(mol/cm^2)**	**Corrected** k_s **(s^{-1})**
n = 3	0.06960	0.08156	0.4605	1.4626	1.1351×10^{-9}	1.5029
n = 5	0.07050	0.07631	0.4802	1.4270	1.4163×10^{-9}	1.4325
n = 8	0.07343	0.07264	0.5027	1.4221	1.6006×10^{-9}	1.4235
n = 10	0.06990	0.07096	0.4962	1.3712	1.8873×10^{-9}	1.0558
n = 13	0.05040	0.05211	0.4917	0.9978	1.3845×10^{-9}	0.8698

Table S2. Parameters obtained by fitting the data shown in Figure 3d according to the equivalent electrical circuit of the inset of Figure 3d

Poly(**Ru1**)$_n$	R_S/Ω cm^2	C_{dl}/μF S^{n-1}	n	R_{ct}/Ω cm^2
n = 3	138.2	362.6	0.6553	229.2
n = 5	117.4	176.7	0.7501	400.2
n = 8	141.5	147.7	0.7277	848.7
n = 10	198.9	54.8	0.7646	1158
n = 13	176.5	115.9	0.8250	2015

Table S3. The comparisons of photocurrent density (*J*) and the IPCE values of Ru complex-based films on ITO

Films	Film-forming technique	*J* (mA cm^{-2})	IPCE(%)	Ref.
(poly**Ru1**)$_8$	EP [a]	11.4[b]	0.127	This work
(poly**Ru2**)$_3$	EP	9.22[b]	0.605	S1
(poly**Ru3**)$_1$	EP	10.7[c]	/	S2
(poly**Ru4**)$_1$	EP	1.36[c]	/	S3
(**Ru5**/PB)$_8$	LB	4.50[d]	/	S4
(GO/**Ru6**)$_1$	ESA [a]	2.68[c]	-0.038	S5
(GO/**Ru7**)$_1$	ESA	1.43[c]	0.04	S6

[a] EP: electropolymerization, ESA: electrostatic self-assembly

[b] -0.4 V vs. SCE containing quinhydrone in 0.1 M Bu_4NPF_6

[c] 0 V vs. SCE

[d] -0.5 V vs. SCE

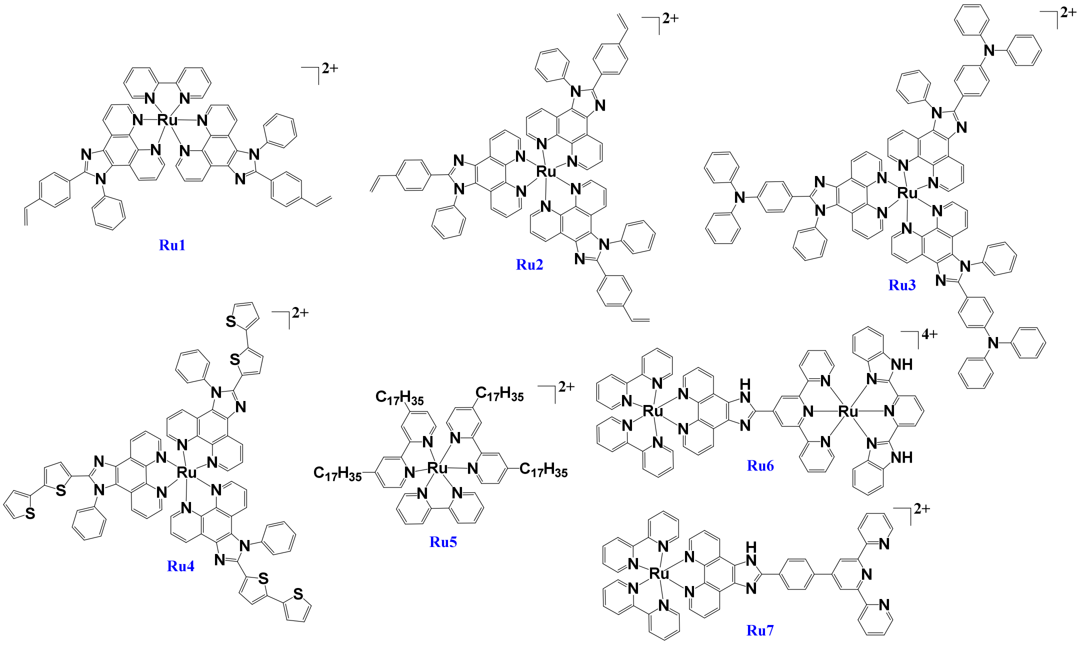

Table S4. Selected PEC ORR for H_2O_2 parameters for organic film modified electrodes

Films	H_2O_2 Productivity	TON	FE_{max}		E_{onset}/V	Experiment condition	Ref.
Poly(**Ru5**)$_8$	2.63 mol/cm^2 (10 h)	1933	34%	PEC	0.54	pH=7 Na_2SO_4 aqueous solution vs. SCE	This work
Poly(**Ru5**)$_8$	1.18 mol/cm^2 (10 h)	871	31%	PC	0.52	pH=7 Na_2SO_4 aqueous solution vs. SCE	This work
pTTh	2.18 mol/cm^2 (10 h)	1606	41%	PEC	/	pH=7 Na_2SO_4 aqueous solution vs. SCE	This work
pTAPP	0.058 mol/cm^2	5.6 ± 1	50%	PEC	0.74	pH=3 acetate buffer	S7
pCoTAPP	0.37 mol/cm^2	17.5 ± 4	70%	PEC	0.92	pH=3 acetate buffer	S7
PN/Au/PTCDI	124~97 mol/cm^2 (6 h)	/	86%	PEC	0.61	pH=2 Na_2SO_4 aqueous solution vs. Ag/AgCl	S8
PN/Au/EPI	133~99 mol/cm^2 (6 h)	/	86%	PEC	0.68	pH=2 Na_2SO_4 aqueous solution vs. Ag/AgCl	S8
pTTh	~110 mmol L^{-1} (11 h, 9 cm^2)	/	90%	PEC	1.15	pH=12.9 KOH aqueous solution 0.65Vvs. RHE	S9

References

S1. H.J. Yin, C.X. Zhang, T. Yang, D.P. Yan, K.Z. Wang. Oxidative electropolymerization films of a styrene-appending ruthenium complex with highly performed electrochemical, solar photoelectric conversion and photoelectrochemical oxygen reduction properties, Electrochim. Acta 403 (2022) 139672-139686.

S2. H. Wang, H.T. Sun, Y.Y. Zhang, C.C. Zhang, Q.R. Cheng, S.M. Hou, J.H. Liao, K.Z. Wang, Three-dimensional high-rate electropolymerized thin film with exceptionally high photocurrent based on a triphenylamine-containing ruthenium complex, Electrochim. Acta 298 (2019) 265-278.

S3. T. Yang, C.X. Zhang, Y.J. Li, Y.H. Fu, Z.H. Yin, L.H. Gao, K. Z. Wang, 3D electropolymerized thin film based on a thiophene-functionalized Ru(II) complex: electrochemical and photoelectrochemical insights, Inorg. Chem. Front. 6 (2019) 3518-3528.

S4. G.R. Torres, E. Dupart, C. Mingotaud, S. Ravaine, Electrochemical and photoelectrochemical properties of new hybrid Langmuir-Blodgett films containing Prussian blue and a tris(bipyridine) ruthenium derivative, J. Phys. Chem. B 104 (2000) 9487–9490.

S5. T.T. Meng, L.X. Xue, H. Wang, K.Z. Wang, M.A. Haga, pH controllable photocurrent switching and molecular half-subtractor calculation based on a monolayer composite film of a dinuclear Ru(II) complex and graphene oxide, J. Mater. Chem. C 5 (2017) 3390–3396.

S6. W. Yang, Z.B. Zheng, T.T. Meng, K.Z. Wang, Synergistically enhanced photoelectrochemical properties of a layer-by-layer hybrid film based on graphene oxide and a free terpyridyl-grafted ruthenium complex, J. Mater. Chem. A 3 (2015) 3441–3449.

S7. N.U. Day, C.C. Wamser, Poly-tetrakis-5,10,15,20-(4-aminophenyl)porphyrin films as two electron oxygen reduction photoelectrocatalysts for the production of H_2O_2, J. Phys. Chem. C 121 (2017) 11076–11082.

S8. M. Gryszel, A. Markov, M. Vagin, E.D. Głowacki, Organic heterojunction photocathodes for optimized photoelectrochemical hydrogen peroxide production, J. Mater. Chem. A 6 (2018) 24709-24716.

S9. W.J. Fan, B.Q. Zhang, X.Y. Wang, W.G. Ma, D. Li, Z.L. Wang, M. Dupuis, J.Y. Shi, S.J. Liao, C. Li, Efficient hydrogen peroxide synthesis by metal-free polyterthiophene via photoelectrocatalytic dioxygen reduction, Energy Environ. Sci. 13 (2020) 238-245.
